# Supplementary material for: Parenchymal cues define Vegfa-driven venous angiogenesis by activating a sprouting competent venous endothelial subtype
Source: Nat Commun. 2024 Apr 10;15:3118. doi: 10.1038/s41467-024-47434-x (PMC11006894; doi:10.1038/s41467-024-47434-x)
Supplement: Supplementary file 1 — Supplementary Information [file 41467_2024_47434_MOESM1_ESM.pdf]

Supplementary Information

Parenchymal cues define Vegfa driven venous angiogenesis by activating a sprouting competent venous endothelial subtype

Preau et al.

NCOMMS-23-15010C

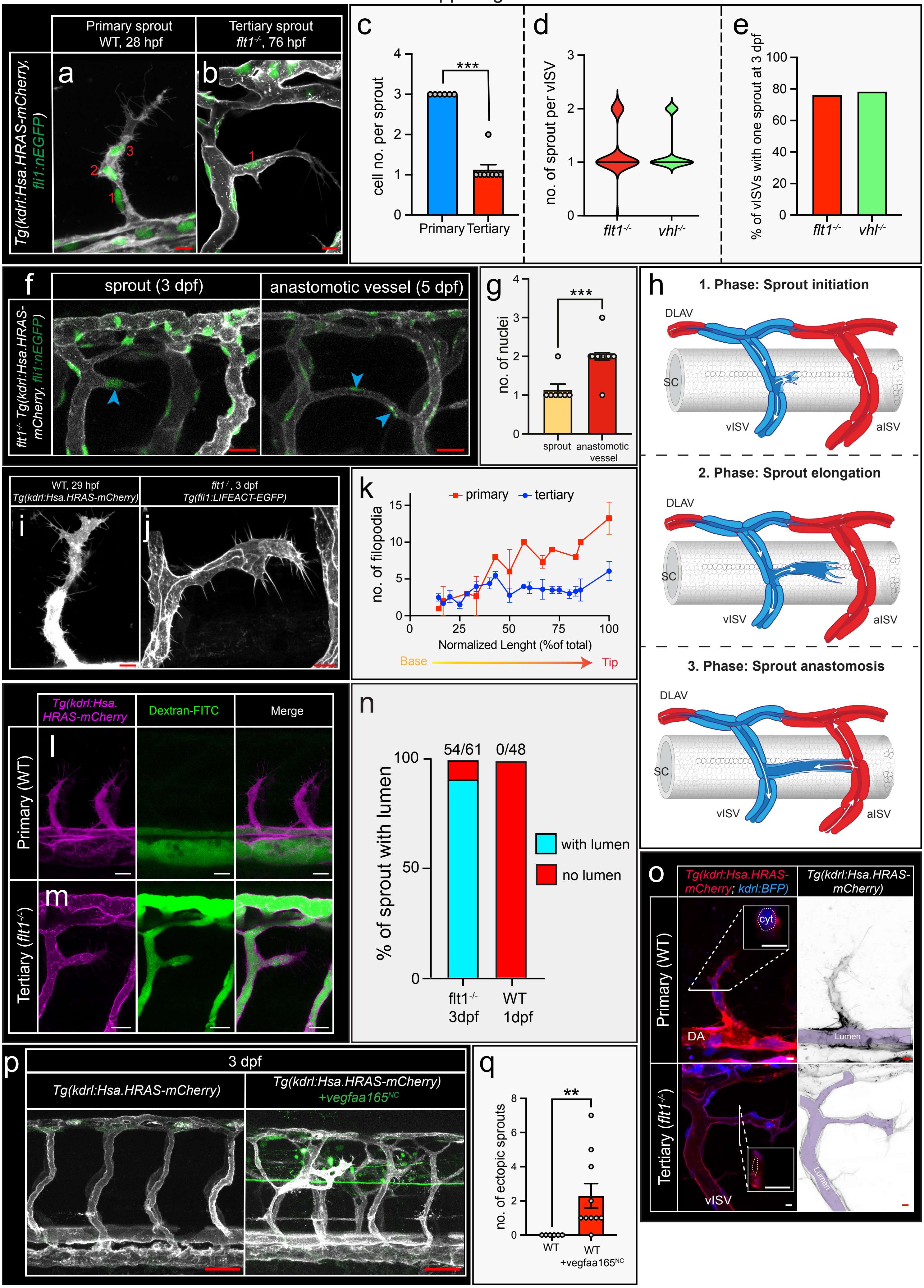

Primary sprout WT, 28 hpf
Tertiary sprout flt1-/-, 76 hpf
Tg(kdrl:Hsa.HRAS-mCherry, fli1:nEGFP)
cell no. per sprout
***
Primary Tertiary
no. of sprout per vISV
flt1-/- vhl-/-
% of vISVs with one sprout at 3 dpf
sprout (3 dpf)
anastomotic vessel (5 dpf)
flt1-/- Tg(kdrl:Hsa.HRAS-mCherry, fli1:nEGFP)
no. of nuclei
sprout anastomotic vessel
1. Phase: Sprout initiation
2. Phase: Sprout elongation
3. Phase: Sprout anastomosis
DLAV
SC
vISV
aISV
WT, 29 hpf Tg(kdrl:Hsa.HRAS-mCherry)
flt1-/-, 3 dpf Tg(fli1:LIFEACT-EGFP)
primary
tertiary
no. of filopodia
Normalized Lenght (%of total)
Base
Tip
Tg(kdrl:Hsa.HRAS-mCherry)
Dextran-FITC
Merge
Primary (WT)
Tertiary (flt1-/-)
54/61
0/48
% of sprout with lumen
with lumen
no lumen
flt1-/- 3dpf
WT 1dpf
Tg(kdrl:Hsa.HRAS-mCherry; kdrl:BFP)
Tg(kdrl:Hsa.HRAS-mCherry)
cyt
DA
Lumen
3 dpf
Tg(kdrl:Hsa.HRAS-mCherry)
Tg(kdrl:Hsa.HRAS-mCherry) +vegfaa165NC
**
no. of ectopic sprouts
WT
WT +vegfaa165NC

Supplement Figure 1. Tertiary sprouts are lumenized structures supported by pericytes and carry blood plasma.

(**a**) Confocal images of *Tg(kdrl:Hsa.HRAS-mCherry; fli1:nEGFP)* showing a primary sprout at 28 hpf in the trunk of zebrafish embryo, EC nuclei in green.
(**b**) Confocal images of *Tg(kdrl:Hsa.HRAS-mCherry; fli1:nEGFP)* showing a tertiary sprout in *flt1*$^{-/-}$ mutant zebrafish embryo at 76 hpf, EC nuclei in green.
(**c**) EC numbers in primary and tertiary sprouts. Mean ± s.e.m., Two-sided Mann-Whitney U test, primary: n=6 sprouts from 6 embryos; tertiary: n=8 sprouts from 8 embryos.
(**d**) Number of tertiary sprouts per venous ISV in *flt1*$^{-/-}$ and *vhl*$^{-/-}$ mutants. Violin Plot with dotted line showing the median; *flt1*$^{-/-}$: n=76 veins from 39 embryos; *vhl*$^{-/-}$: n=14 veins from 7 embryos.
(**e**) Percentage of venous ISVs with one tertiary sprout in *flt1*$^{-/-}$ and *vhl*$^{-/-}$ mutants. *flt1*$^{-/-}$: n=76 veins from 39 embryos; *vhl*$^{-/-}$: n=14 veins from 7 embryos.
(**f**) Confocal imaging of *Tg(kdrl:Hsa.HRAS-mCherry; fli1:nEGFP)* showing tertiary sprouts during sprouting (left panel, 3 dpf) and after anastomosis (right panel, 5 dpf). EC nuclei (blue arrowheads) in green. Note: After anastomosis, the tertiary sprout is colonized by an additional EC.
(**g**) EC numbers in sprout and upon anastomosis formation. Mean ± s.e.m., Two-sided Mann-Whitney U test; sprout: n=7 sprouts from 4 embryos, anastomotic: n=16 vessel segments from 6 embryos.
(**h**) Schematic representation of the tertiary sprouting and remodelling process. In the dorsal part of venous ISV, spinal cord derived cues induce the formation of a lumenized endothelial tip cell (L-Tip cell, upper panel). The cell subsequently enlarges and elongates in the direction of a neighboring artery (aISV, middle panel). The cell contacts the aISV and anastomoses with it (lower panel). Arrows indicate the blood flow direction.
(i) Confocal images of *Tg(kdrl:Hsa.HRAS-mCherry)* showing filopodia in a primary sprout at 29 hpf.
(**j**) Confocal images of *Tg(fli1:LIFEACT-EGFP)* showing filopodia in a tertiary sprout at 3 dpf.
(**k**) Quantification of filopodia distribution in primary (red) and tertiary (blue) sprouts. Note: In primary sprouts, the majority of the filopodia is located at the tip. Mean ± s.e.m.; primary: n=6 sprouts; tertiary: n=14 sprouts.
(**l**) Injection of FITC labelled dextran into *Tg(kdrl:Hsa.HRAS-mCherry)* to image blood plasma distribution shows absence of blood plasma in primary sprouts consistent with absence of lumenization.
(**m**) Injection of FITC labelled dextran into *Tg(kdrl:Hsa.HRAS-mCherry)* to image blood plasma distribution shows blood plasma in the tertiary sprout consistent with lumenization during sprouting.
(**n**) Proportion of lumenized primary and tertiary sprouts based on FITC-dextran injections, n=48 primary sprouts and n=61 tertiary sprouts.
(**o**) Confocal images of primary (top) and tertiary (bottom) sprouts using *Tg(kdrl:Hsa.HRAS-mCherry)* (red) to label the EC cell membrane and *Tg(kdrl:TagBFP)* (blue) to label the EC cytoplasm (left panels). Boxed area: Optical cross-section at indicated position in the sprout. Note: Cross sections of tertiary sprouts lack endothelial cytoplasm in the center of the section indicating that they have a lumen. Right panels: Pseudo colouring (purple) of the vessel lumen.
(**p**) Confocal images of *Tg(kdrl:Hsa.HRAS-mCherry)* showing trunk vasculature in WT (left panel) and WT upon neuronal *vegfaa*^{165} overexpression (right panel) at 3 dpf.
(q) Quantification of ectopic sprouts upon neuronal *vegfaa*^{165} overexpression. Mean ± s.e.m., Two-sided Mann-Whitney U test; WT: n=6 embryos; neuronal *vegfaa*^{165} overexpression: n=10 embryos.
Scale bar indicates 10 µm in a, b, I, j; 20 µm in f, i, l, m; 5 µm in o; 50 µm in p. vISV, venous intersegmental vessel; aISV, arterial intersegmental vessel; DLAV, dorsal longitudinal anastomotic vessel; SC, spinal cord; cyt, cytoplasm. *, p<0.05, **, p<0.01 and ***, p<0.001. Source data are provided as a Source Data file.

Suppl. Fig. 2

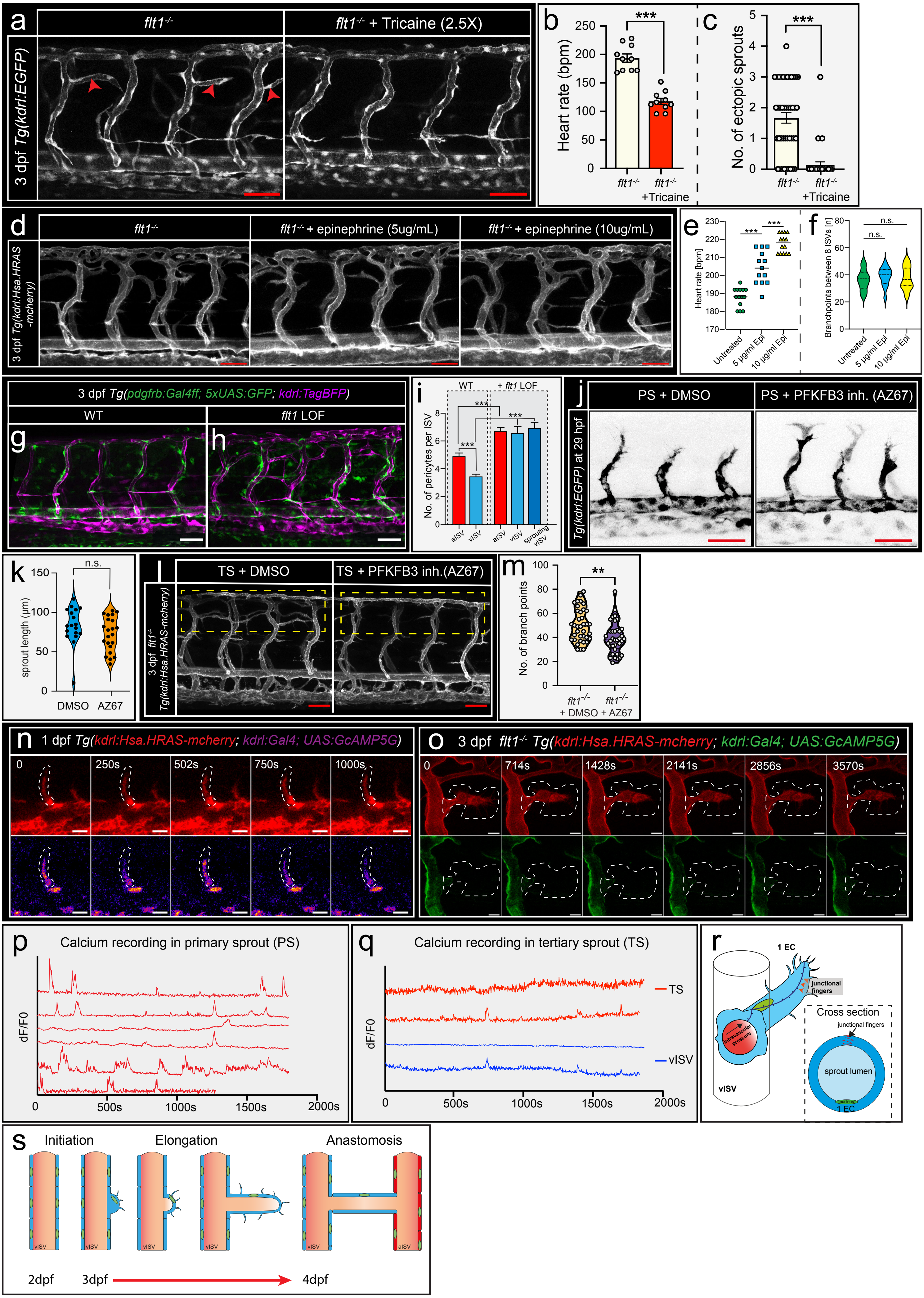

a
flt1-/-
flt1-/- + Tricaine (2.5X)
3 dpf Tg(kdrl:EGFP)
b
Heart rate (bpm)
***
flt1-/-
flt1-/- +Tricaine
c
No. of ectopic sprouts
***
flt1-/-
flt1-/- +Tricaine
d
flt1-/-
flt1-/- + epinephrine (5ug/mL)
flt1-/- + epinephrine (10ug/mL)
3 dpf Tg(kdrl:Hsa.HRAS-mcherry)
e
Heart rate [bpm]
***
Untreated
5 µg/ml Epi
10 µg/ml Epi
f
Branchpoints between 8 ISVs [n]
n.s.
3 dpf Tg(pdgfrb:Gal4ff; 5xUAS:GFP; kdrl:TagBFP)
WT
flt1 LOF
g
h
i
+ flt1 LOF
No. of pericytes per ISV
aISV
vISV
sprouting vISV
j
PS + DMSO
PS + PFKFB3 inh. (AZ67)
Tg(kdrl:EGFP) at 29 hpf
k
sprout length (µm)
DMSO
AZ67
l
TS + DMSO
TS + PFKFB3 inh.(AZ67)
3 dpf flt1-/- Tg(kdrl:Hsa.HRAS-mcherry)
m
No. of branch points
**
flt1-/- + DMSO
flt1-/- + AZ67
n
1 dpf Tg(kdrl:Hsa.HRAS-mcherry; kdrl:Gal4; UAS:GcAMP5G)
0
250s
502s
750s
1000s
o
3 dpf flt1-/- Tg(kdrl:Hsa.HRAS-mcherry; kdrl:Gal4; UAS:GcAMP5G)
714s
1428s
2141s
2856s
3570s
p
Calcium recording in primary sprout (PS)
dF/F0
500s
1000s
1500s
2000s
q
Calcium recording in tertiary sprout (TS)
TS
vISV
r
1 EC
junctional fingers
intravascular pressure
vISV
Cross section
sprout lumen
nucleus
s
Initiation
Elongation
Anastomosis
vISV
aISV
2dpf
3dpf
4dpf

Supplement Figure 2. Comparison of Tertiary sprouting with Primary sprouting.

(**a**) Confocal images of *Tg(kdrl:EGFP)* showing trunk vasculature in *flt1*$^{-/-}$ mutant (left panel) or *flt1*$^{-/-}$ mutant treated with tricaine to reduce trunk perfusion (right panel) at 3 dpf. Note loss of ectopic venous sprouting upon reducing flow.
(**b**) Heart rate in untreated or tricaine treated *flt1*$^{-/-}$ mutant. Note: Tricaine treatment reduces the heart rate by 50%. Mean ± s.e.m., Two-sided Unpaired t-test with Welch correction, n=10 embryos per condition.
(**c**) Ectopic sprout number in indicated scenario. Note: reducing blood flow almost completely abolishes tertiary sprouting. Mean ± s.e.m., Two-sided Mann-Whitney U test; *flt1*$^{-/-}$: n=43 embryos, *flt1*$^{-/-}$ + Tricaine: 35 embryos.
(**d**) Confocal images of *Tg(kdrl:Hsa.HRAS-mCherry)* showing trunk vasculature in *flt1*$^{-/-}$ mutant (left panel) or *flt1*$^{-/-}$ mutant treated with epinephrine to increase perfusion (center and right panels) at 3 dpf.
(e) Heart rate in indicated scenario. Note: epinephrine treatment increases heart rate. Untreated: n=14 embryos; 5 µg/mL Epi: n=13 embryos; 10 µg/mL Epi: n=14 embryos.
(**f**) Quantification of branching complexity at ROI in indicated scenario. Violin Plot, dotted lines showing median and interquartiles; Two-sided Unpaired t-test with Welch correction; Untreated: n=18 embryos; 5 µg/mL Epi: n=14 embryos; 10 µg/mL Epi: n= 16 embryos.
(**g,h**) Confocal images showing pericyte distribution (green) around trunk blood vessels (purple) in WT (g) and *flt1* loss of function scenario (h) at 3 dpf.
(**i**) Quantification of pericyte distribution around trunk aIVS, vISV and tertiary sprouts. Mean ± s.e.m., Two-sided Mann-Whitney U test, WT: n=110 ISVs from 29 embryos; *flt1* LOF: n=90 ISVs from 20 embryos.
(**j**) Confocal images of *Tg(kdrl:EGFP)* showing primary sprouts in DMSO (left) or AZ67 (right) treated embryos at 29 hpf.
(**k**) Primary sprout length in DMSO (left) or PFKFB3 inhibitor (AZ67, right) treated embryos. Note: glycolysis inhibition has no significant effect on primary sprouting. Violin Plot, dotted lines showing median and interquartiles; Two-sided Unpaired t-test; DMSO: n=18 ISVs from 9 embryos, AZ67: n=22 ISVs from 11 embryos.
(**l**) Confocal images of *Tg(kdrl:Hsa.HRAS-mCherry)* showing trunk vasculature of *flt1*$^{-/-}$ mutant treated with DMSO (left) or PFKFB3 inhibitor (AZ67, right) at 3 dpf. Yellow box indicates ROI.
(**m**) Quantification of branching complexity at ROI in *flt1*$^{-/-}$ mutant treated with DMSO or AZ67. Note: glycolysis inhibition reduces tertiary sprouting events. Violin Plot, dotted lines showing median and interquartiles; Two-sided Unpaired t-test with Welch correction; *flt1*$^{-/-}$: n=23 embryos; *flt1*$^{-/-}$ + AZ67: n=29 embryos.
(**n**) *In vivo* calcium recording using indicated reporter line during primary sprouting at 1 dpf.
(**o**) *In vivo* calcium recording using indicated reporter line during tertiary sprouting at 3 dpf.
(**p, q**) Quantification of Ca^{2+} dynamics in primary (p) and tertiary (q) sprouts. Note: Primary sprouting is associated with calcium waves, whereas no calcium waves were observed in tertiary sprouting. Shown is normalized fluorescence (dF/F0) in the calcium reporter line. Each curve represents the fluorescence variations at the level of a single ROI. Primary: n=6 sprouts; Tertiary: n=2 sprouts; vISV: n=2 ROI.
(**r**) Schematic representation of the lumenized tip cell (L-Tip Cell) in the tertiary sprout. The peripheral domains of the L-Tip Cell connect with each other *via* finger like junctions (arrowhead) to form a lumenized vessel segment that carries blood plasma.
(**s**) Schematic representation of the tertiary sprouting process. The L-Tip Cell in the venous domain responds to angiogenic cues, enlarges, forms a lumen and expands into a functional anastomotic vessel connecting to its lateral aISV.
Scale bar indicates 50 µm in a, d, g, h, j, l; 20 µm in n and o. LOF, loss of function; PS, primary sprout; TS, tertiary sprout; aISV, arterial intersegmental vessel; vISV, venous intersegmental vessel; EC, endothelial cell. *, *p*<0.05, **, *p*<0.01 and ***, *p*<0.001. Source data are provided as a Source Data file.

Suppl. Fig. 3

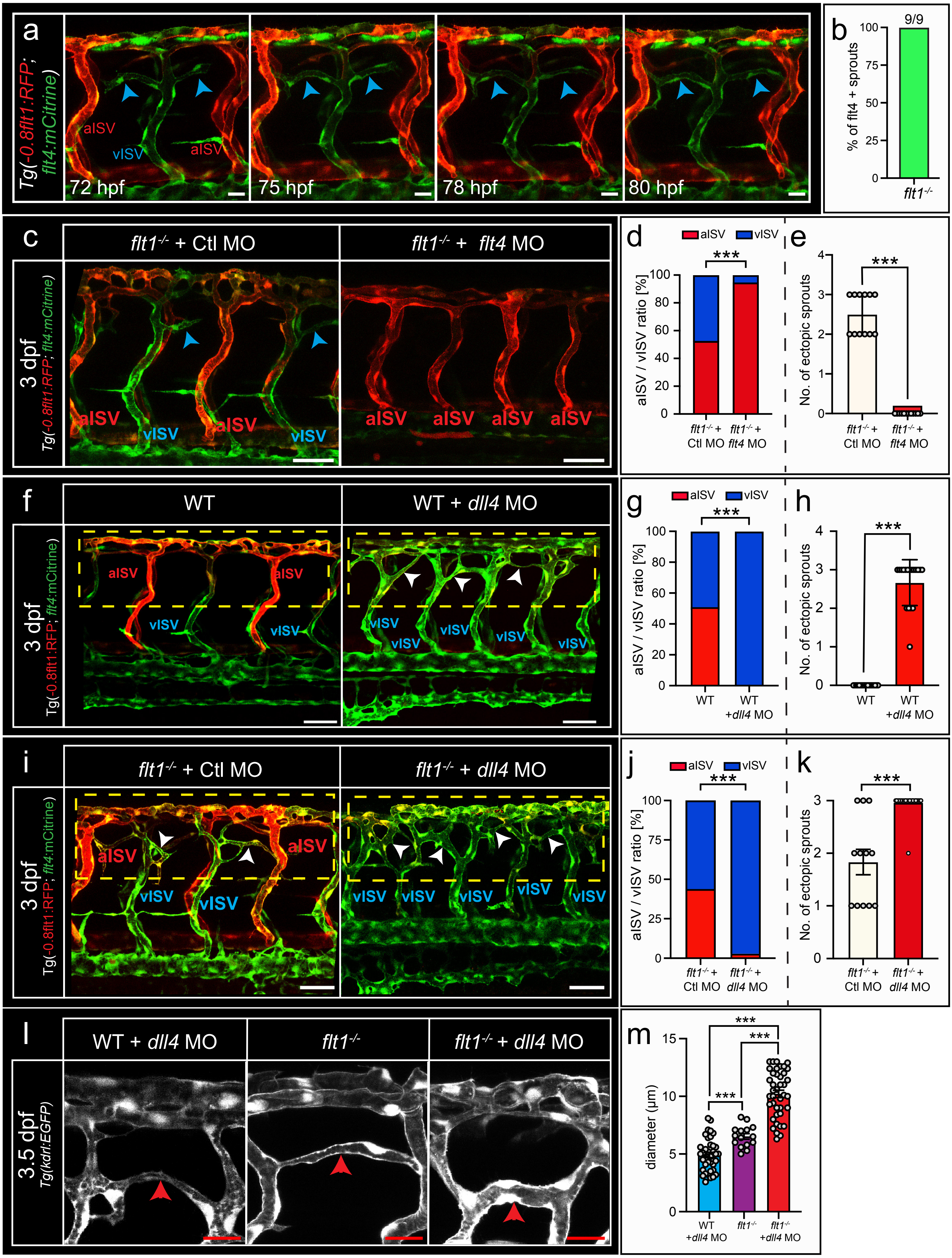

a
Tg(-0.8flt1:RFP; flt4:mCitrine)
aISV
vISV
aISV
72 hpf
75 hpf
78 hpf
80 hpf
b
9/9
% of flt4 + sprouts
flt1-/-
c
flt1-/- + Ctl MO
flt1-/- + flt4 MO
3 dpf
Tg(-0.8flt1:RFP; flt4:mCitrine)
aISV
vISV
d
aISV
vISV
***
aISV / vISV ratio [%]
flt1-/- + Ctl MO
flt1-/- + flt4 MO
e
***
No. of ectopic sprouts
f
WT
WT + dll4 MO
3 dpf
Tg(-0.8flt1:RFP; flt4:mCitrine)
g
h
WT
WT +dll4 MO
i
flt1-/- + Ctl MO
flt1-/- + dll4 MO
j
k
l
WT + dll4 MO
flt1-/-
flt1-/- + dll4 MO
3.5 dpf
Tg(kdrl:EGFP)
m
diameter (µm)
WT +dll4 MO
flt1-/-
flt1-/- +dll4 MO

Supplement Figure 3. Tertiary sprouts emanate from veins

(**a**) Still images of time-lapse imaging during tertiary sprout elongation and anastomosis formation in *Tg(-0.8flt1:RFP; flt4:mCitrine)*. Arteries are marked by *Tg(-0.8flt1:RFP) (red),* veins by *Tg(flt4:mCitrine) (green)*. Note: Tertiary sprouts (blue arrowheads) express the venous marker *flt4* (green) during the whole sprouting process until anastomosis formation.

(**b**) Proportion of tertiary sprout expressing *flt4* based on time lapse as displayed in (a). Note: All tertiary sprouts express the venous marker *flt4*. n=9 sprouts from 4 time-lapses on 4 embryos.

(**c**) Confocal images of *Tg(-0.8flt1:RFP; flt4:mCitrine)* showing trunk vasculature in *flt1*$^{-/-}$ mutant injected with control (left panel) or *flt4* targeting morpholino (right panel) at 3 dpf. Note: *flt4* loss of function results in a trunk vasculature consisting almost exclusively of arterial ISVs and lacking tertiary sprouts. Blue arrowheads point on tertiary sprouts.

(**d**) Arterial to venous ISV ratio for indicated scenario. Mean, Fisher's exact test; Ctl MO: n=12 embryos, *flt4* MO: n=9 embryos.

(**e**) Number of ectopic spouts for indicated scenario. Mean ± s.e.m., Two-sided Mann-Whitney U test; Ctl MO: n=12 embryos, *flt4* MO: n=9 embryos.

(**f**) Confocal images showing trunk vasculature in WT (left panel) and WT injected with a *dll4* morpholino (right panel) in *Tg(-0,8flt1:RFP; flt4:mCitrine*) at 3 dpf. Arteries are shown in red, veins in green. Note: *dll4* loss of function results in a trunk vasculature consisting mainly of veins, and formation of ectopic sprouts at the level of the spinal cord. Arrowheads point on tertiary sprouts.

(**g**) Arterial to venous ISV ratio for indicated scenario. Note: more veins upon loss of *dll4*. Mean, Fisher's exact test, n=18 embryos per condition.

(**h**) Quantification of ectopic sprouting in WT and *dll4* morphants. Mean ± s.e.m., Two-sided Mann-Whitney U test, n=18 embryos per condition.

(**i**) Confocal images showing trunk vasculature in *flt1*$^{-/-}$ mutant injected with control (left panel) or *dll4* targeting morpholino (right panel) at 3 dpf. Yellow dotted box indicates region of interest. Arrowheads point on tertiary sprouts.

(**j**) Arterial to venous ISV ratio for indicated scenario. Note: *dll4* loss of function induces an increase of vein proportion. Mean, Fisher's exact test; Ctl MO n=12 embryos; *dll4* MO n=27 embryos.

(**k**) Quantification of ectopic sprouting in *flt1*$^{-/-}$ mutant and *flt1*$^{-/-}$ mutant upon *dll4* loss of function. Mean ± s.e.m., Two-sided Mann-Whitney U test; Ctl MO n=12 embryos; *dll4* MO n=27 embryos.

(**l**) Confocal images in *Tg(kdrl:EGFP)* showing ectopic vessel morphology (red arrowhead) in WT injected with *dll4* targeting morpholino (left), *flt1*$^{-/-}$ mutant (middle) and *flt1*$^{-/-}$ mutant injected with *dll4* targeting morpholino (right) at 3.5 dpf.

(**m**) Quantification of ectopic vessel diameter for indicated scenario. Mean ± s.e.m., Two-sided Unpaired t-test; WT + *dll4* MO: n=36 sprouts from 21 embryos, *flt1*$^{-/-}$: n=15 sprouts from 14 embryos, *flt1*$^{-/-}$ + dll4MO: n=40 sprouts from 34 embryos.

Scale bar indicates 20 µm in a and l; 50 µm in c, f and i. aISV, arterial intersegmental vessel; vISV, venous intersegmental vessel; MO, morpholino. *, $p<0.05$, **, $p<0.01$ and ***, $p<0.001$. Source data are provided as a Source Data file.

Suppl. Fig. 4

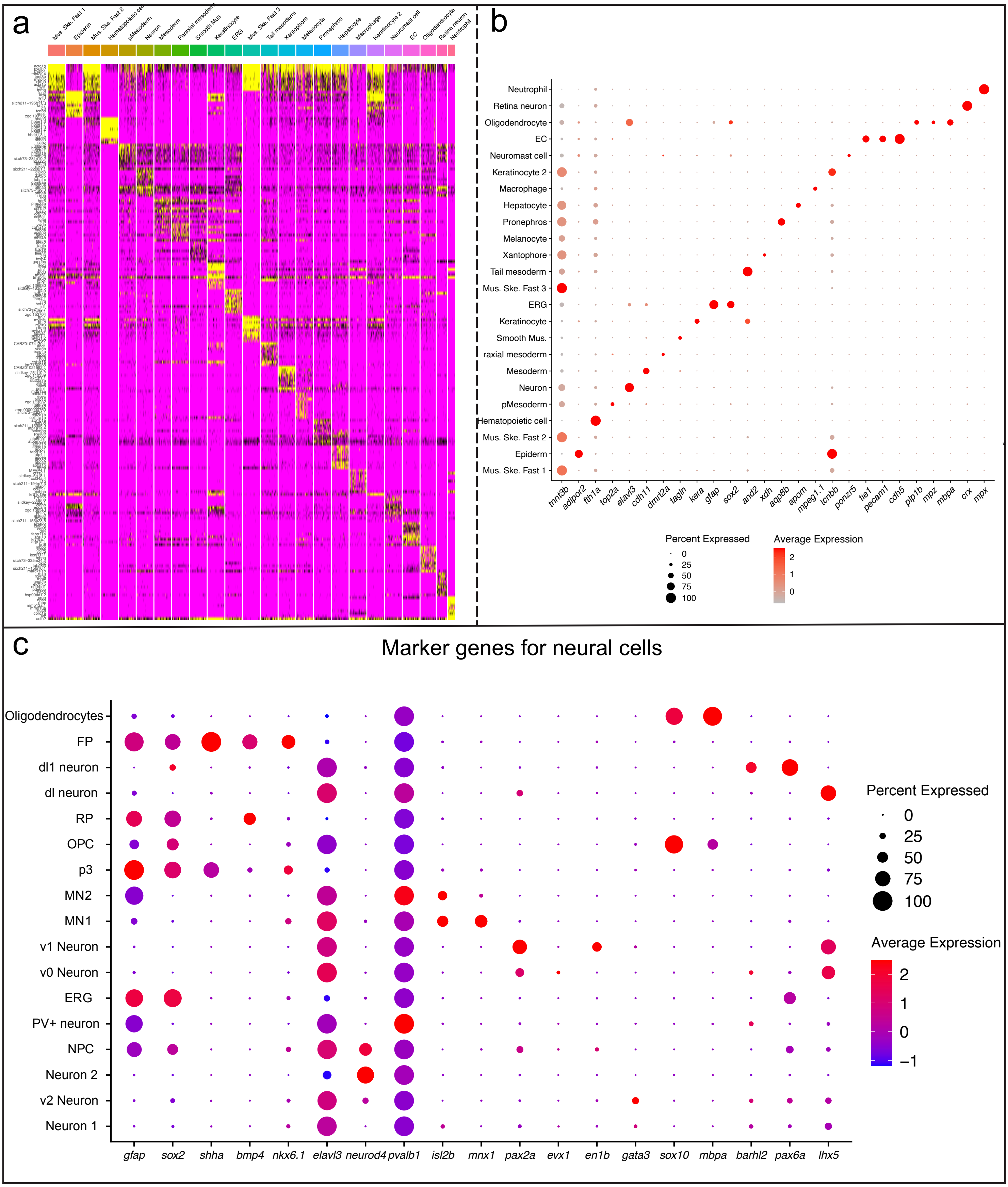
a
Mus. Ske. Fast 1
Epiderm
Mus. Ske. Fast 2
Hematopoietic cell
pMesoderm
Neuron
Mesoderm
Paraxial mesoderm
Smooth Mus
Keratinocyte
ERG
Mus. Ske. Fast 3
Tail mesoderm
Xantophore
Melanocyte
Pronephros
Hepatocyte
Macrophage
Keratinocyte 2
Neuromast cell
EC
Oligodendrocyte
Retina neuron
Neutrophil
b
Neutrophil
Retina neuron
Oligodendrocyte
EC
Neuromast cell
Keratinocyte 2
Macrophage
Hepatocyte
Pronephros
Melanocyte
Xantophore
Tail mesoderm
Mus. Ske. Fast 3
ERG
Keratinocyte
Smooth Mus.
raxial mesoderm
Mesoderm
Neuron
pMesoderm
Hematopoietic cell
Mus. Ske. Fast 2
Epiderm
Mus. Ske. Fast 1
tnnt3b adipor2 fth1a top2a elavl3 cdh11 dmrt2a tagln kera gfap sox2 and2 xdh aqp8b apom mpeg1.1 tcnbb ponzr5 tie1 pecam1 cdh5 plp1b mpz mbpa crx mpx
Percent Expressed
0
25
50
75
100
Average Expression
2
1
0
c
Marker genes for neural cells
Oligodendrocytes
FP
dI1 neuron
dI neuron
RP
OPC
p3
MN2
MN1
v1 Neuron
v0 Neuron
ERG
PV+ neuron
NPC
Neuron 2
v2 Neuron
Neuron 1
gfap sox2 shha bmp4 nkx6.1 elavl3 neurod4 pvalb1 isl2b mnx1 pax2a evx1 en1b gata3 sox10 mbpa barhl2 pax6a lhx5
Percent Expressed
0
25
50
75
100
Average Expression
2
1
0
−1

Supplement Figure 4. Expression of cell type marker genes in the zebrafish embryo trunk.

(**a**) Heatmap indicating the top 10 marker genes for each cell type.
(**b**) Dot plot showing the expression of marker genes for the trunk cell types. Dot size represents the percentage of cells expressing the gene, and colours represent the average expression of the gene within a cluster.
(**c**) Dot plot showing the expression of marker genes for the neural cell types. Dot size represents the percentage of cells expressing the gene, and colours represent the average expression of the gene within a cluster.

Suppl. Fig. 5

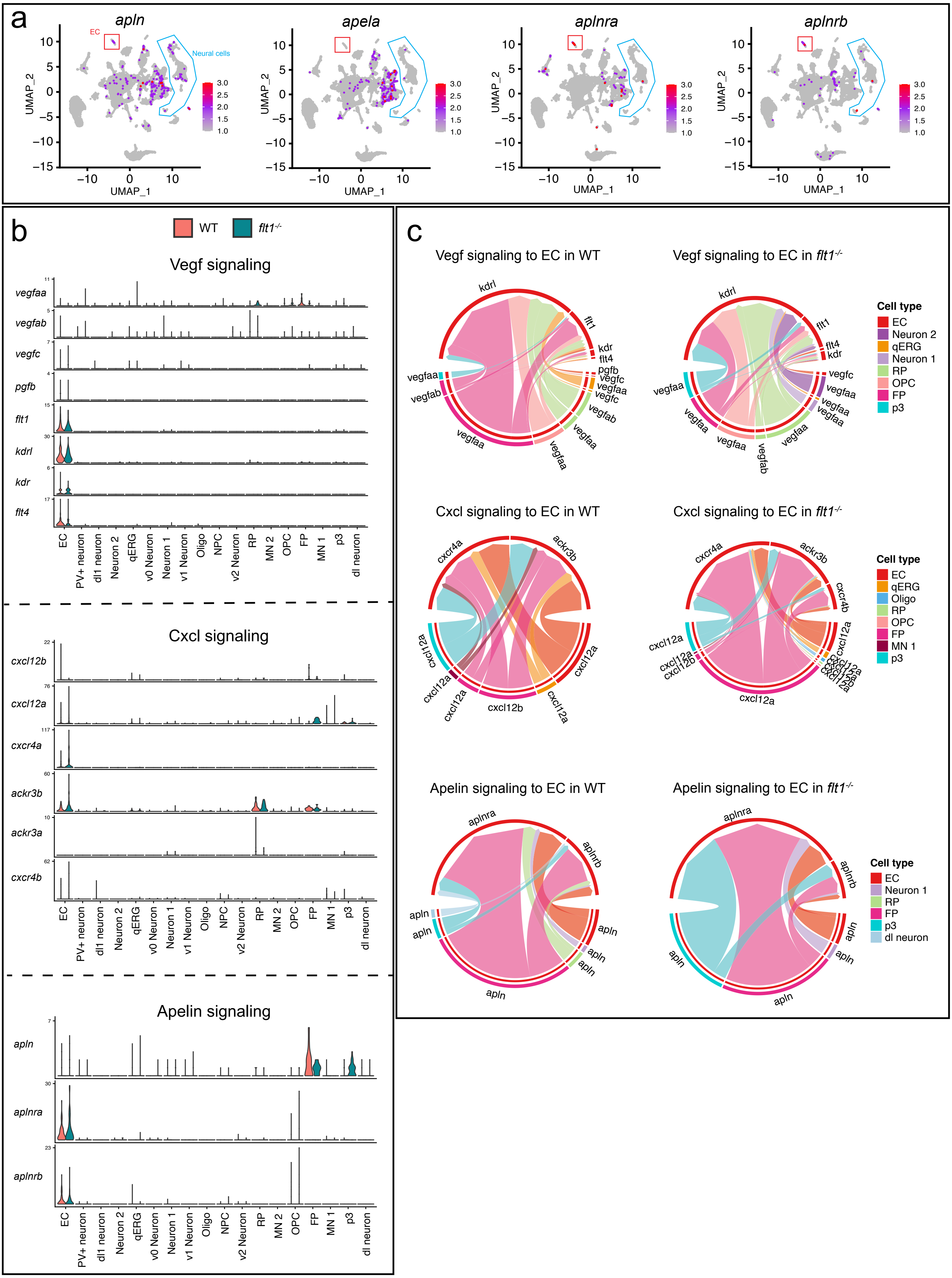

a
apln
apela
aplnra
aplnrb
EC
Neural cells
UMAP_1
UMAP_2
b
WT
flt1-/-
Vegf signaling
vegfaa
vegfab
vegfc
pgfb
flt1
kdrl
kdr
flt4
Cxcl signaling
cxcl12b
cxcl12a
cxcr4a
ackr3b
ackr3a
cxcr4b
Apelin signaling
apln
aplnra
aplnrb
EC
PV+ neuron
dI1 neuron
Neuron 2
qERG
v0 Neuron
Neuron 1
v1 Neuron
Oligo
NPC
v2 Neuron
RP
MN 2
OPC
FP
MN 1
p3
dI neuron
c
Vegf signaling to EC in WT
Vegf signaling to EC in flt1-/-
Cxcl signaling to EC in WT
Cxcl signaling to EC in flt1-/-
Apelin signaling to EC in WT
Apelin signaling to EC in flt1-/-
Cell type
EC
Neuron 2
qERG
Neuron 1
RP
OPC
FP
p3
Oligo
MN 1
dI neuron

Supplement Figure 5. Expression of Apelin, Vegf and Cxcl signalling pathway components at the neurovascular interface.

(a) Feature Plot showing the expression of *apelin*, *apela*, *aplnra* and *aplnrb* in zebrafish trunk.
(**b**) Violin Plot showing the expression of Vegf (top), Cxcl (middle) and Apelin (bottom) signalling pathway components in EC and neural cells used to perform the Cell Chat analyses.
(**c**) Chord diagram showing neural to endothelial cell communication for Vegf (top), Cxcl (middle) and Apelin (bottom) pathways in WT and *flt1*$^{-/-}$ mutant.

Suppl. Fig. 6

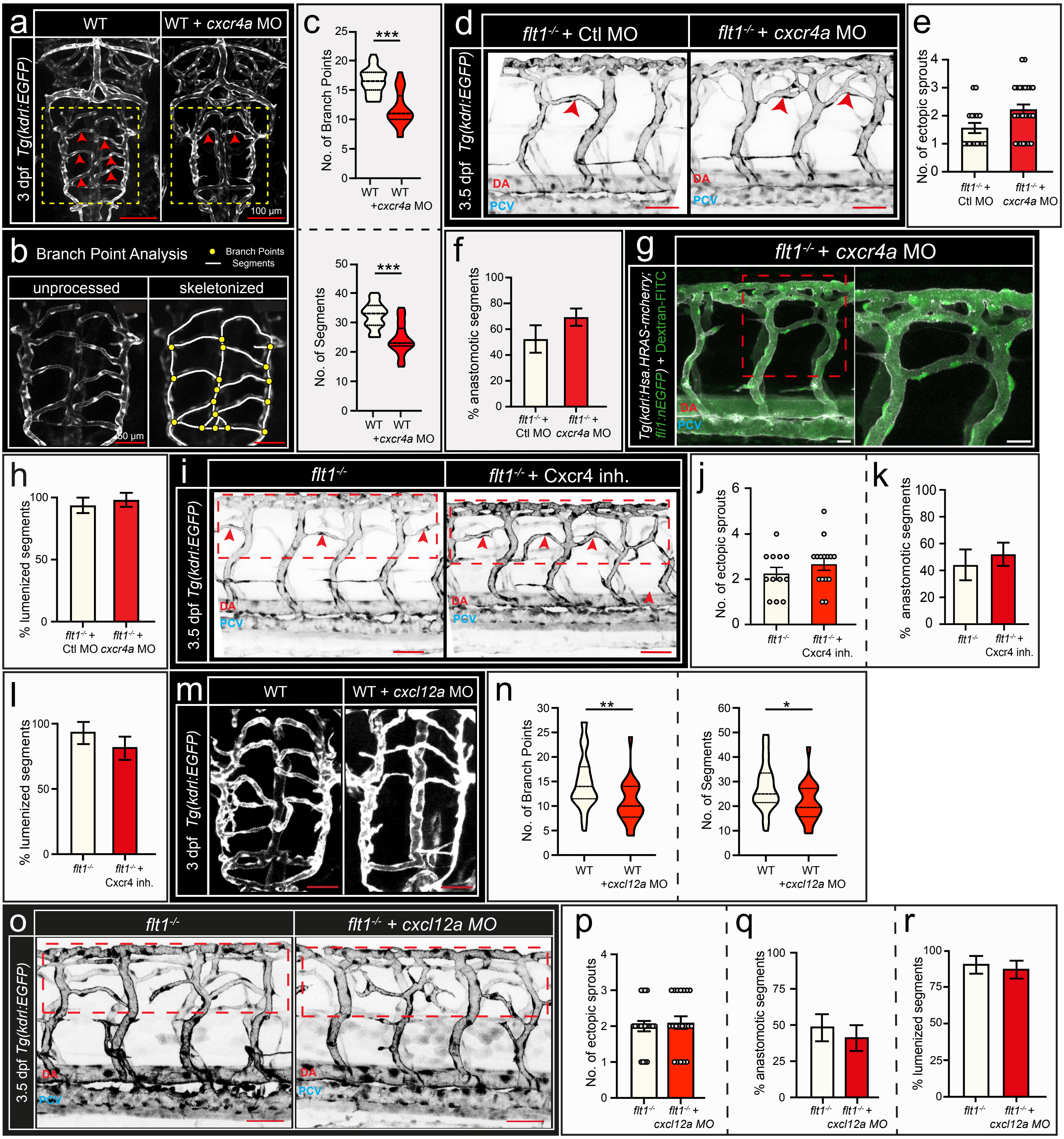

a
WT
WT + cxcr4a MO
3 dpf Tg(kdrl:EGFP)
100 µm
b Branch Point Analysis
Branch Points
Segments
unprocessed
skeletonized
50 µm
c
No. of Branch Points
No. of Segments
***
WT
WT +cxcr4a MO
d
flt1-/- + Ctl MO
flt1-/- + cxcr4a MO
3.5 dpf Tg(kdrl:EGFP)
DA
PCV
e
No. of ectopic sprouts
flt1-/- + Ctl MO
flt1-/- + cxcr4a MO
f
% anastomotic segments
g
flt1-/- + cxcr4a MO
Tg(kdrl:Hsa.HRAS-mcherry; fli1:nEGFP) + Dextran-FITC
h
% lumenized segments
i
flt1-/-
flt1-/- + Cxcr4 inh.
j
k
l
m
WT + cxcl12a MO
3 dpf Tg(kdrl:EGFP)
n
**
*
WT +cxcl12a MO
o
flt1-/- + cxcl12a MO
p
q
r

Supplement Figure 6. Cxcl signalling is not required for tertiary sprouting in *flt1$^{-/-}$* mutant.

(**a**) Confocal images showing hindbrain vasculature in 3 dpf WT (left panel) and WT injected with *cxcr4a* targeting morpholino (right panel). Red arrowheads indicate lumenized anastomotic connections. Note the significant reduction of lumenized anastomotic connections between central artery and basilar artery upon loss of *cxcr4a*. Dotted yellow box indicates the ROI used for (**b**) Semi-automated hindbrain vascular network analysis using Image J. Branch point and number of vessel segments were determined as indicated.
(**c**) Quantification of hindbrain vessel branching complexity upon loss of *cxcr4a*. Violin plots showing the branchpoint and segment number distribution, lines represent the median (dashed lines) and the interquartiles (dotted lines), Two-sided Mann-Whitney U test, WT: n=28 embryos and WT+ *cxcr4a* MO: n=27 embryos. Note: Loss of *cxcr4a* significantly reduced hindbrain angiogenic remodelling.
(**d**) Confocal images showing trunk vasculature in *flt1$^{-/-}$* mutant injected with control (left panel) or *cxcr4a* targeting morpholino (right panel) at 3.5 dpf. Arrowheads indicate lumenized anastomotic connections between venous and arterial ISVs.
(**e**) Quantification of ectopic sprouting at the level of the spinal cord for indicated scenario. Mean ± s.e.m.; Ctl MO: n=19 embryos, *cxcr4a* MO: n=21 embryos.
(**f**) Anastomotic vISV to aISV connections at the level of the spinal cord for the indicated scenario. Mean ± s.e.m.; Ctl MO: n=19 embryos, *cxcr4a* MO: n=21 embryos.
(**g**) Injection of FITC-dextran to visualize blood plasma distribution in the trunk vasculature of *flt1$^{-/-}$* mutants injected with *cxcr4a* targeting morpholino (left panel) at 3.5 dpf; boxed area at higher magnification in right panel. Note: The ectopic anastomotic connections are perfused upon loss of *cxcr4a*.
(**h**) Proportion of lumenized segments for indicated scenarios. Mean ± s.e.m.; Ctl MO: n=19 embryos, *cxcr4a* MO: n=21 embryos.
(**i**) Confocal images of trunk vasculature in *flt1$^{-/-}$* mutant treated with vehicle (left panel) or Cxcr4 inhibitor (right panel) between 2.5 to 3.5 dpf. Red arrowheads indicate anastomotic connections between venous and arterial ISVs.
(**j, k, l**) Quantification of ectopic sprout number (j), anastomotic connections between vISV and aISV (k) and lumenization (l) for the indicated scenarios. Note: Sprouting, lumenization and vISV-aISV connections were not affected by inhibition of Cxcr4. Mean ± s.e.m.; DMSO: n=12 embryos; Cxcr4 inh.: n=16 embryos.
(**m**) Confocal images of *Tg(kdrl:EGFP)* showing hindbrain vasculature in 3 dpf WT (left panel) and WT injected with *cxcl12a* targeting morpholino (right panel). Note: reduction of anastomotic connections between central artery and basilar artery upon loss of *cxcl12a*.
(**n**) Quantification of hindbrain vessel branching complexity upon loss of *cxcl12a* based on images in (m). Violin plot showing the branchpoint distribution, lines represent the median (dashed lines) and the interquartiles (dotted lines); Two-sided Mann-Whitney U test for the No. of Branchpoints and t-test with Welch's correction for the No. of segments; WT: n= 29 embryos, WT + *cxcl12a* MO: n= 22 embryos.
(**o**) Confocal images of *Tg(kdrl:EGFP)* showing trunk vasculature in *flt1$^{-/-}$* mutant injected with control (left panel) or *cxcl12a* targeting morpholino (right panel) at 3.5 dpf.
(**p, q, r**) Quantification of ectopic sprout number (p), anastomotic connections between vISV and aISV (q) and lumenization (r) for the indicated scenarios. Note: Sprouting, lumenization and vISV-aISV connections were not affected by loss of *cxcl12a*. Mean ± s.e.m.; Two-sided Mann-Whitney U test; n=20 embryos per conditions.
Scale bar indicates 100 µm in a; 50 µm in b, d, i, m, o; 20 µm in g. MO, morpholino; DA, dorsal aorta; PCV, Posterior cardinal vein; inh., inhibitor. *, p<0.05, **, p<0.01 and ***, p<0.001. Source data are provided as a Source Data file.

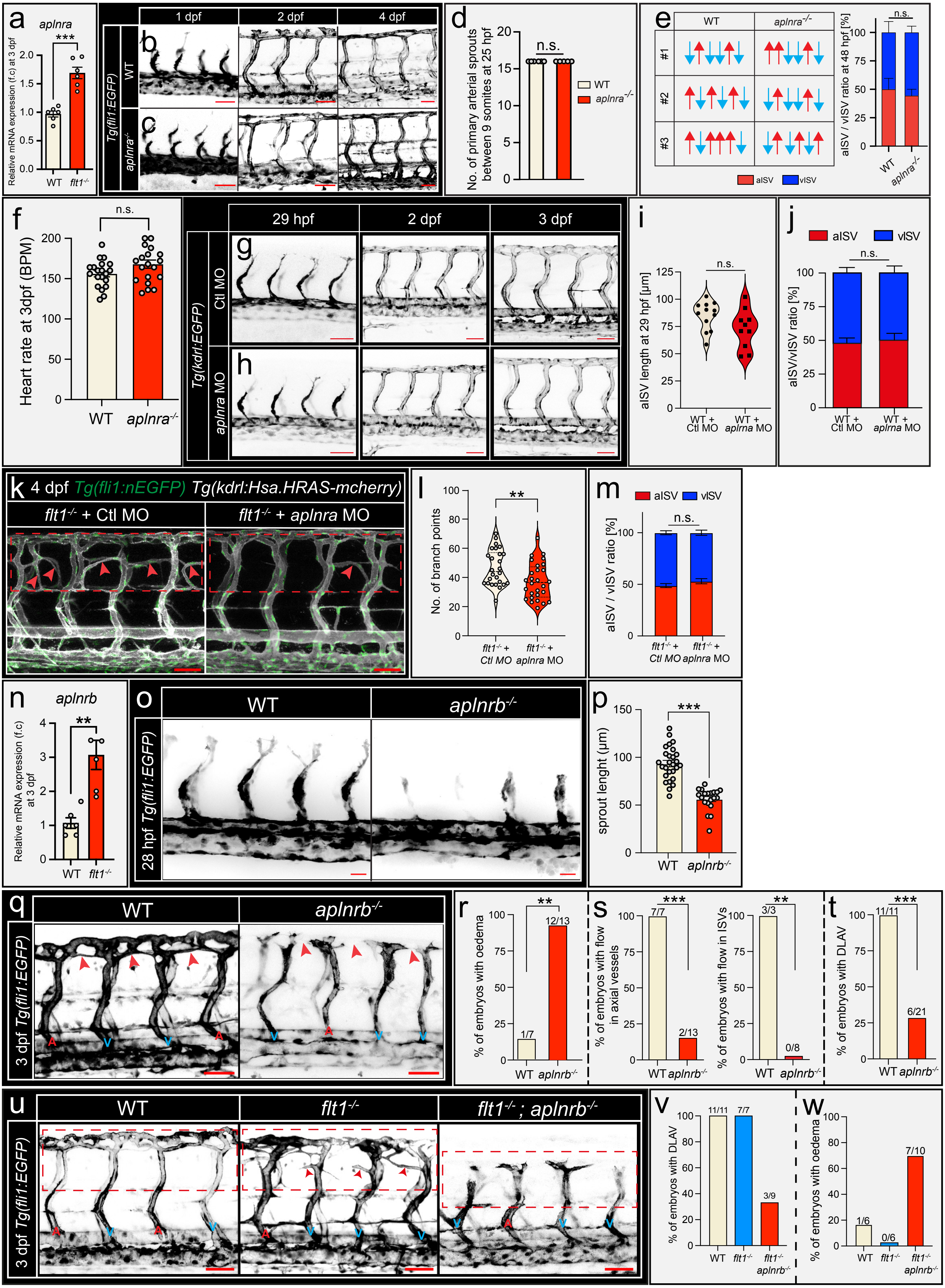

a aplnra
Relative mRNA expression (f.c) at 3 dpf
WT flt1-/-
***
b c
1 dpf 2 dpf 4 dpf
Tg(fli1:EGFP)
WT
aplnra-/-
d
No. of primary arterial sprouts between 9 somites at 25 hpf
n.s.
WT
aplnra-/-
e
WT aplnra-/-
#1 #2 #3
aISV vISV
aISV / vISV ratio at 48 hpf [%]
n.s.
f
Heart rate at 3dpf (BPM)
n.s.
WT aplnra-/-
29 hpf 2 dpf 3 dpf
Tg(kdrl:EGFP)
g Ctl MO
h aplnra MO
i
aISV length at 29 hpf [µm]
n.s.
WT + Ctl MO WT + aplrna MO
j
aISV vISV
aISV/vISV ratio [%]
n.s.
WT + Ctl MO WT + aplnra MO
k 4 dpf Tg(fli1:nEGFP) Tg(kdrl:Hsa.HRAS-mcherry)
flt1-/- + Ctl MO flt1-/- + aplnra MO
l
No. of branch points
**
flt1-/- + Ctl MO flt1-/- + aplnra MO
m
aISV vISV
aISV / vISV ratio [%]
n.s.
flt1-/- + Ctl MO flt1-/- + aplnra MO
n aplnrb
Relative mRNA expression (f.c) at 3 dpf
**
WT flt1-/-
o
WT aplnrb-/-
28 hpf Tg(fli1:EGFP)
p
sprout lenght (µm)
***
WT aplnrb-/-
q
WT aplnrb-/-
3 dpf Tg(fli1:EGFP)
A V
r
% of embryos with oedema
**
1/7 12/13
WT aplnrb-/-
s
% of embryos with flow in axial vessels
***
7/7 2/13
WT aplnrb-/-
% of embryos with flow in ISVs
**
3/3 0/8
WT aplnrb-/-
t
% of embryos with DLAV
***
11/11 6/21
WT aplnrb-/-
u
WT flt1-/- flt1-/- ; aplnrb-/-
3 dpf Tg(fli1:EGFP)
v
% of embryos with DLAV
11/11 7/7 3/9
WT flt1-/- flt1-/- aplnrb-/-
w
% of embryos with oedema
1/6 0/6 7/10
WT flt1-/- flt1-/- aplnrb-/-

Supplement Figure 7. Vascular phenotypes of *aplnra*$^{-/-}$ and *aplnrb*$^{-/-}$ mutants.

(**a**) *Aplnra* expression in WT and *flt1*$^{-/-}$ mutant embryos at 3 dpf. Note: Significantly increased *aplnra* expression in *flt1*$^{-/-}$. Mean ± s.e.m., Two-sided Unpaired t-test with Welch's correction; n=6 samples of 20 pooled embryos at 3 dpf.
(**b,c**) Confocal images showing trunk vasculature in WT (b) and *aplnra*$^{-/-}$ mutant (c) at indicated time points.
(**d**) Quantification of primary artery sprouting events in WT and *aplnra*$^{-/-}$ mutant based on images in (b,c). Note: Loss of *aplnra* had no impact on primary sprouting. Mean ± s.e.m., Two-sided Mann-Withney U test, WT: n=9 embryos; *aplnra*$^{-/-}$: n=5 embryos.
(**e**) ISV flow pattern at 48 hpf; venous flow direction in blue (vISV); arterial flow direction in red (aISV) – in WT (left panel) and *aplnra*$^{-/-}$ mutants (right panel) and quantification of arterial ISV to venous ISV ratio in WT and *aplnra*$^{-/-}$ mutant. Mean ± s.e.m., Fisher's exact test; WT: n=3 embryos; *aplnra*$^{-/-}$: n=3 embryos.
(**f**) Heart rate (bpm) in WT and *aplnra*$^{-/-}$ mutant. Mean ± s.e.m., Two-sided Unpaired t-test with Welch's correction; n=10 embros per condition.
(**g,h**) Confocal images showing ISV development in WT injected with a control (g) or *aplnra* targeting morpholino (h) at indicated timepoints.
(**i**) Quantification of aISV length based on images in (g,h). Violin plot shows length distribution, Two-sided Mann-Whitney U test, Ctl MO: n=10 ISVs from 5 embryos, *aplnra* MO: n=10 ISVs from 5 embryos.
(**j**) Arterial to venous ISV ratio for indicated scenario. Note: knock-down of *aplnra* in WT had no significant impact on arterial-venous specification of ISVs. Mean ± s.e.m., Fisher's exact test, Ctl MO: n=11 embryos, *aplnra* MO: n=10 embryos.
(**k**) Confocal images showing trunk vasculature in *flt1*$^{-/-}$ mutant injected with control (left panel) or *aplnra* targeting morpholino (right panel) at 4 dpf. Red dotted box indicates region of interest; arrowheads indicate ectopic vessels.
(**l**) Quantification of branching complexity for indicated scenario. Violin plot shows the branchpoint distribution, lines represent the median (dashed lines) and the interquartile (dotted lines), Two-sided Mann-Whitney U test, Ctl MO: n=19 embryos; *aplnra* MO: n=26 embryos.
(**m**) Arterial to venous ISV ratio for indicated scenario. Note: Knock-down of *aplnra* in *flt1*$^{-/-}$ had no significant impact on arterial-venous specification of ISVs. Mean ± s.e.m., Fisher's exact test, Ctl MO: n=19 embryos, *aplnra* MO: n=26 embryos.
(**n**) *aplnrb* expression in WT and *flt1*$^{-/-}$ mutant embryo at 3 dpf. Note: Significantly increased *aplnrb* expression in *flt1*$^{-/-}$. Mean ± s.e.m.;Two-sided Unpaired t-test with Welch's correction; n=6 samples of 20 pooled embryos at 3 dpf.
(**o**) Confocal images of *Tg(fli1:EGFP)* showing trunk vasculature in 28 hpf WT (left) and *aplnrb*$^{-/-}$ mutant (right) during primary sprouting.
(**p**) Quantification of primary sprout length in WT and *aplnrb*$^{-/-}$ mutant based on images in (o). Mean ± s.e.m.,;Two-sided Mann-Whitney U test; WT: n=27 ISVs from 6 embryos, *aplnrb*$^{-/-}$: n= 20 ISVs from 4 embryos.
(**q**) Confocal images of *Tg(fli1:EGFP)* showing trunk vasculature in 3 dpf WT (left) and *aplnrb*$^{-/-}$ mutant (right); arrowheads indicate DLAV position. Note: *aplnrb*$^{-/-}$ mutants lack the DLAV and do not form a perfused trunk vessel network.
(**r**) Frequency of oedema in WT and *aplnrb*$^{-/-}$ mutant. Percentage, Fisher's exact test; WT: n= 7 embryos, *aplnrb*$^{-/-}$: n= 13 embryos.
(**s**) Percentage of embryos displaying adequate axial vessel (left panel) or ISV (right panel) perfusion in WT and *aplnrb*$^{-/-}$ mutant. Percentage, Fisher's exact test; WT: n= 7 embryos, *aplnrb*$^{-/-}$: n= 13 embryos for axial vessel flow and WT: n= 3 embryos, *aplnrb*$^{-/-}$: n= 8 embryos for ISV flow.
(**t**) Percentage of embryos displaying normal DLAV formation in WT and *aplnrb*$^{-/-}$ mutant. Percentage, Fisher's exact test; WT: n= 11 embryos, *aplnrb*$^{-/-}$: n= 21 embryos.
(**u**) Confocal imaging of *Tg(fli1:EGFP)* showing trunk vasculature in 3 dpf WT (left), *flt1*$^{-/-}$ (middle) and *flt1*$^{-/-}$; *aplnrb*$^{-/-}$ (right). Red dotted box indicates the region of interest; arrowheads indicate ectopic vessels. Note: the *flt1*$^{-/-}$; *aplnrb*$^{-/-}$ double mutants lack a DLAV, ISVs do not extend properly in dorsal direction and ectopic sprouts are not present.
(**v, w**) Quantification of DLAV formation (v) and oedema frequency (w) for indicated scenario. WT: n=11 embryos, *flt1*$^{-/-}$: n=7 embryos, *flt1*$^{-/-}$; *aplnrb*$^{-/-}$: n=9 embryos for DLAV and WT: n=6 embryos, *flt1*$^{-/-}$: n=6 embryos, *flt1*$^{-/-}$; *aplnrb*$^{-/-}$: n= 10 embryos.
Scale bar indicates 50 µm in b, c, g, h, k, q, u; 20 µm in o. A, arterial intersegmental vessel; V, venous intersegmental vessel; MO, morpholino; BPM, beat per minute. *, *p*<0.05, **, *p*<0.01 and ***, *p*<0.001. Source data are provided as a Source Data file.

Suppl. Fig. 8

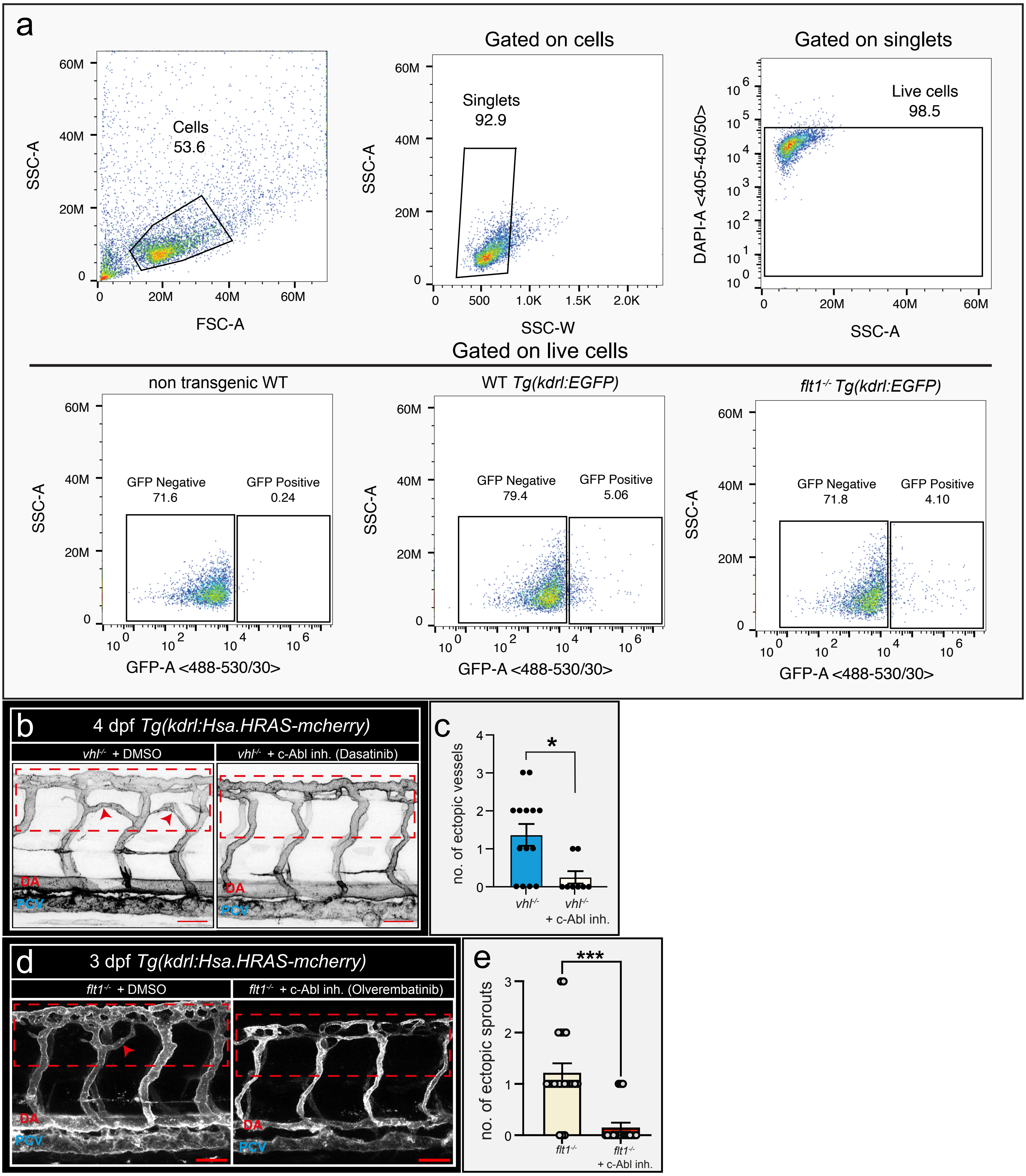

a
Gated on cells
Gated on singlets
Cells
53.6
SSC-A
FSC-A
Singlets
92.9
SSC-W
Live cells
98.5
DAPI-A <405-450/50>
Gated on live cells
non transgenic WT
GFP Negative
71.6
GFP Positive
0.24
WT Tg(kdrl:EGFP)
GFP Negative
79.4
GFP Positive
5.06
flt1-/- Tg(kdrl:EGFP)
GFP Negative
71.8
GFP Positive
4.10
GFP-A <488-530/30>
b
4 dpf Tg(kdrl:Hsa.HRAS-mcherry)
vhl-/- + DMSO
vhl-/- + c-Abl inh. (Dasatinib)
DA
PCV
c
no. of ectopic vessels
vhl-/-
vhl-/- + c-Abl inh.
d
3 dpf Tg(kdrl:Hsa.HRAS-mcherry)
flt1-/- + DMSO
flt1-/- + c-Abl inh. (Olverembatinib)
e
no. of ectopic sprouts
flt1-/-
flt1-/- + c-Abl inh.

Supplement Figure 8. FACS strategy for endothelial cell sorting and c-Abl inhibition.

(**a**) Gating strategy for FACS sorting endothelial cells from WT and *flt1*$^{-/-}$ *Tg(kdrl:EGFP)* embryos.
(**b**) Confocal images showing trunk vasculature in *vhl*$^{-/-}$ mutant treated with DMSO (left) or with the c-Abl inhibitor Dasatinib (right) at 4 dpf. Red dotted box indicates the region of interest; arrowheads indicate lumenized anastomotic vessel segments. Note: Dasatinib reduces ectopic branching.
(**c**) Quantification of ectopic vessels for indicated scenario. Mean ± s.e.m., Two-sided Mann-Whitney U test, DMSO: n=14 embryos and Dasatinib: n=8 embryos.
(**d**) Confocal images of *Tg(kdrl:Hsa.HRAS-mCherry)* showing trunk vasculature in *flt1*$^{-/-}$ embryos treated with DMSO (left) or with the c-Abl inhibitor Olverembatinib (right) at 3 dpf. Red dotted box indicates the region of interest; arrowheads indicate ectopic sprouts.
(**e**) Quantification of ectopic sprouts for the indicated scenario. Mean ± s.e.m., Two-sided Mann-Whitney U test, DMSO: n=22 embryos, Olverembafatinib: n=19 embryos.
*, *p*<0.05, **, *p*<0.01 and ***, *p*<0.001. Source data are provided as a Source Data file.

Suppl. Fig. 9

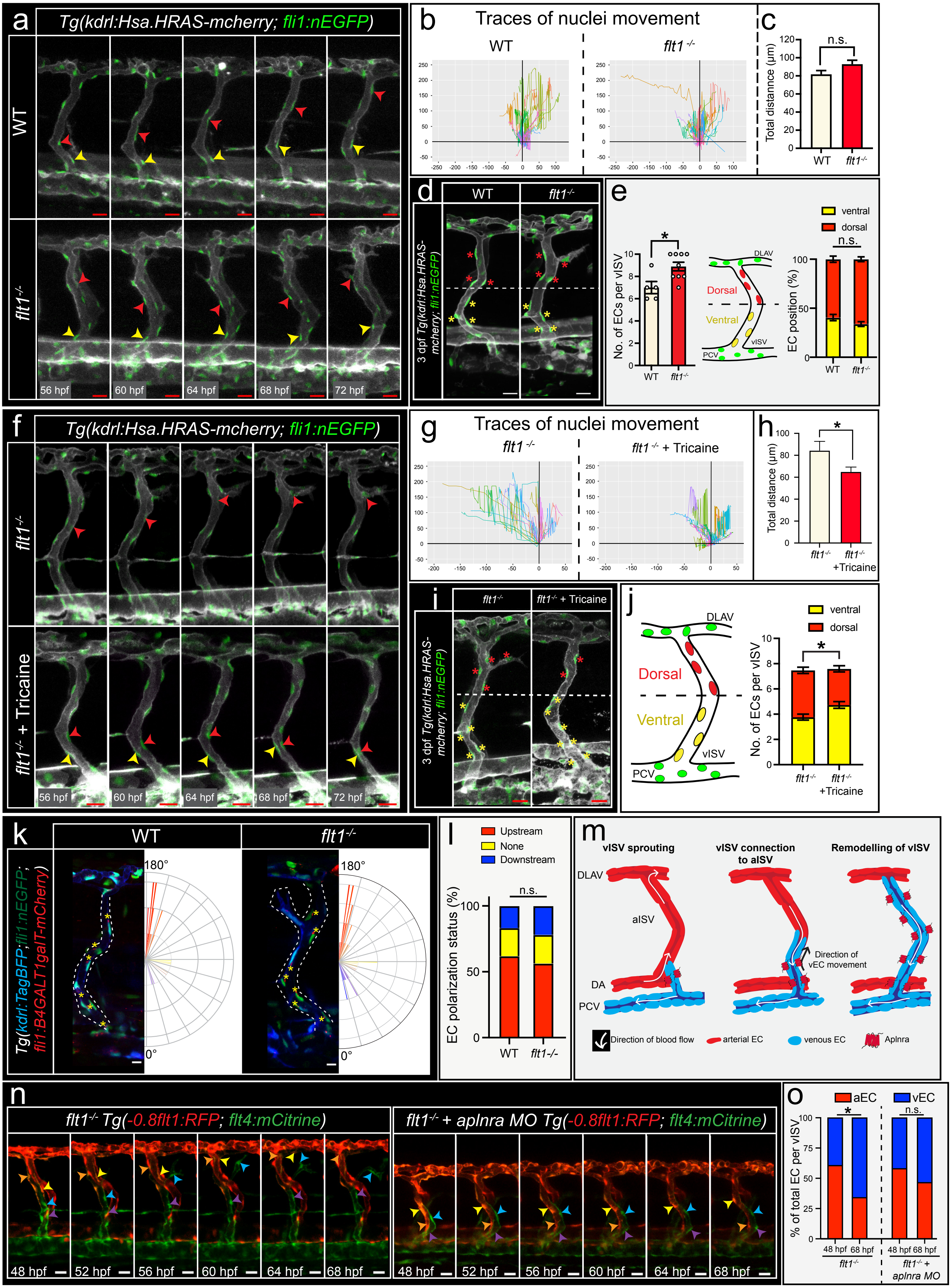

a
Tg(kdrl:Hsa.HRAS-mcherry; fli1:nEGFP)
WT
flt1-/-
56 hpf
60 hpf
64 hpf
68 hpf
72 hpf
b
Traces of nuclei movement
WT
flt1 -/-
c
n.s.
Total distance (µm)
WT
flt1-/-
d
WT
flt1-/-
3 dpf Tg(kdrl:Hsa.HRAS-mcherry; fli1:nEGFP)
e
ventral
dorsal
No. of ECs per vISV
DLAV
Dorsal
Ventral
vISV
PCV
EC position (%)
n.s.
f
Tg(kdrl:Hsa.HRAS-mcherry; fli1:nEGFP)
flt1-/-
flt1-/- + Tricaine
g
Traces of nuclei movement
flt1 -/-
flt1 -/- + Tricaine
h
Total distance (µm)
flt1-/-
flt1-/- +Tricaine
i
flt1-/-
flt1-/- + Tricaine
3 dpf Tg(kdrl:Hsa.HRAS-mcherry; fli1:nEGFP)
j
DLAV
Dorsal
Ventral
vISV
PCV
ventral
dorsal
No. of ECs per vISV
flt1-/-
flt1-/- +Tricaine
k
WT
flt1-/-
Tg(kdrl:TagBFP;fli1:nEGFP; fli1:B4GALT1galT-mCherry)
180°
0°
l
Upstream
None
Downstream
EC polarization status (%)
n.s.
WT
flt1-/-
m
vISV sprouting
vISV connection to aISV
Remodelling of vISV
DLAV
aISV
DA
PCV
Direction of vEC movement
Direction of blood flow
arterial EC
venous EC
Aplnra
n
flt1-/- Tg(-0.8flt1:RFP; flt4:mCitrine)
flt1-/- + aplnra MO Tg(-0.8flt1:RFP; flt4:mCitrine)
48 hpf
52 hpf
56 hpf
60 hpf
64 hpf
68 hpf
o
aEC
vEC
n.s.
% of total EC per vISV
48 hpf
68 hpf
flt1-/-
flt1-/- + aplnra MO

Supplement Figure 9. Ventral-dorsal migration of EC in venous ISVs of WT and *flt1*$^{-/-}$.

(a) Still images of *Tg(kdrl:Hsa.HRAS-mCherry; fli1:nEGFP)* time-lapse showing endothelial cell movements in WT (top) and *flt1*$^{-/-}$ (bottom) at indicated time points. Arrowheads (yellow, red) indicate individual endothelial cell nuclei and their ventral to dorsal migration trajectory in the vISV.
(b) Traces of individual endothelial cell movements in WT (left) and *flt1*$^{-/-}$ (right). Scale in pixels; WT: n=35 venous EC from 7 embryos, *flt1*$^{-/-}$: n=32 venous EC from 9 embryos.
(c) Quantification of endothelial migration distance in vISVs for the indicated scenario. Mean ± s.e.m., Two-sided Unpaired t-test with Welch's correction; WT: n=35 venous EC from 7 embryos, *flt1*$^{-/-}$: n=32 venous EC from 9 embryos.
(**d**) Confocal images of *Tg(kdrl:Hsa.HRAS-mCherry; fli1:nEGFP)* showing endothelial cell distribution in vISVs of WT (left panel) and *flt1*$^{-/-}$ (right panel) at 3 dpf. Red asterisks mark ECs in the dorsal domain; yellow asterisks mark ECs in the ventral domain.
(**e**) Quantification of venous EC number (left panel) and position (right panel) in WT and *flt1*$^{-/-}$ mutant. Note: *flt1*$^{-/-}$ mutants have a higher number of EC per vISV but their positions are not changed compared to WT. Mean ± s.e.m., Two-sided Mann-Whitney U test for the total EC number and Fisher's exact test for the EC position, WT: n=5 vISVs from 3 embryos, *flt1*$^{-/-}$: n=9 vISVs from 4 embryos.
(**f**) Still images of *Tg(kdrl:Hsa.HRAS-mCherry; fli1:nEGFP)* time-lapse showing endothelial cell nuclei movements in *flt1*$^{-/-}$ (top) and in *flt1*$^{-/-}$ embryos treated with tricaine (bottom) at indicated time points. Arrowheads indicate endothelial cell nuclei and their migration trajectory in the vISV.
(**g**) Traces of individual endothelial cell nuclei movements in *flt1*$^{-/-}$ (left) and *flt1*$^{-/-}$ treated with tricaine (right). Scale in pixels; *flt1*$^{-/-}$: n=20 venous EC from 5 embryos, *flt1*$^{-/-}$ + tricaine: n=26 venous EC from 5 embryos.
(**h**) Quantification of endothelial migration distance in vISVs for the indicated scenario. Mean ± s.e.m., Two-sided Mann-Whitney U test, *flt1*$^{-/-}$: n=20 venous EC from 5 embryos, *flt1*$^{-/-}$ + tricaine: n=26 venous EC from 5 embryos. Note: Tricaine treatment reduces venous EC migration.
(**i**) Confocal images in *Tg(kdrl:Hsa.HRAS-mCherry; fli1:nEGFP)* showing endothelial cell distribution in vISVs of *flt1*$^{-/-}$ (left panel) and *flt1*$^{-/-}$ treated with tricaine (right panel) at 3 dpf. Red asterisks mark ECs in the dorsal, yellow asterisks in the ventral domain. Note: Tricaine treatment results in accumulation of ECs in the ventral part of the vISV.
(**j**) Quantification of dorsal-ventral distribution of endothelial cells for the indicated scenario. Mean ± s.e.m, Fischer's exact test, n=22 ISVs from 9 and 11 embryos.
(**k**) Confocal images in *Tg(kdrl:TagBFP; fli1:nEGFP; fli1:B4GALT1galT-mCherry)* and diagram showing the polarization status of individual endothelial cells in vISV for WT embryos (left panel), and *flt1*$^{-/-}$ (right panel). Note: No difference in EC polarization between WT and *flt1*$^{-/-}$.
(**l**) Quantification of endothelial cell polarization events in WT and *flt1*$^{-/-}$. Chi-square test, WT: n=4 vISV from 3 embryos and *flt1*$^{-/-}$: n=7vISV from 3 embryos.
(**m**) Scheme illustrating venous EC migration events during arterio-venous remodelling. After secondary sprouting, the newly formed veins are colonized by venous ECs coming from the PCV. These venous ECs express *aplnra*.
(**n**) Still images from time-lapse of *flt1*$^{-/-}$ or *flt1*$^{-/-}$ injected with *aplnra* MO in *Tg(-0.8flt1:RFP; flt4:mCitrine)* reporter line during vein remodelling process. In *flt1*$^{-/-}$ mutant (left panel), venous ECs (green; blue and purple arrowheads) migrated from the PCV to the DLAV and replaced the arterial ECs (red; yellow and orange arrowheads). Only the venous ECs contribute to tertiary sprout formation. Upon *aplnra* loss of function (right panel), the migration of the venous ECs is reduced and arterial ECs remain in the vISV. We never observed that venous ECs start to express the arterial marker *flt1* (red) or that artery ECs start to migrate into the vISVs. Embryo in right panel was slightly tilted during embedding giving the impression that the fish is smaller but it is not the case.
(**o**) Quantification of the endothelial cell composition of vISV in *flt1*$^{-/-}$ (left panel) and *flt1*$^{-/-}$ upon *aplnra* loss of function (right panel). At 48 hpf, more than half of the ECs in vISV are arterial (red). In *flt1*$^{-/-}$ mutant, at 68 hpf, venous ECs have migrated into the vISV and are now representing the majority of the ECs. In *flt1*$^{-/-}$ upon *aplnra* loss of function, there is reduced vECs migration resulting in higher aECs present in vISV at 68 hpf.
Scale bar indicates 20 µm in a, d, f, i, n; 10 µm in k. DLAV, dorsal longitudinal anastomotic vessels; vISV, venous intersegmental vessel; PCV, posterior cardinal vein; EC, endothelial cell; DA, dorsal aorta; aISV; arterial intersegmental vessel. *, *p*<0.05, **, *p*<0.01 and ***, *p*<0.001. Source data are provided as a Source Data file.

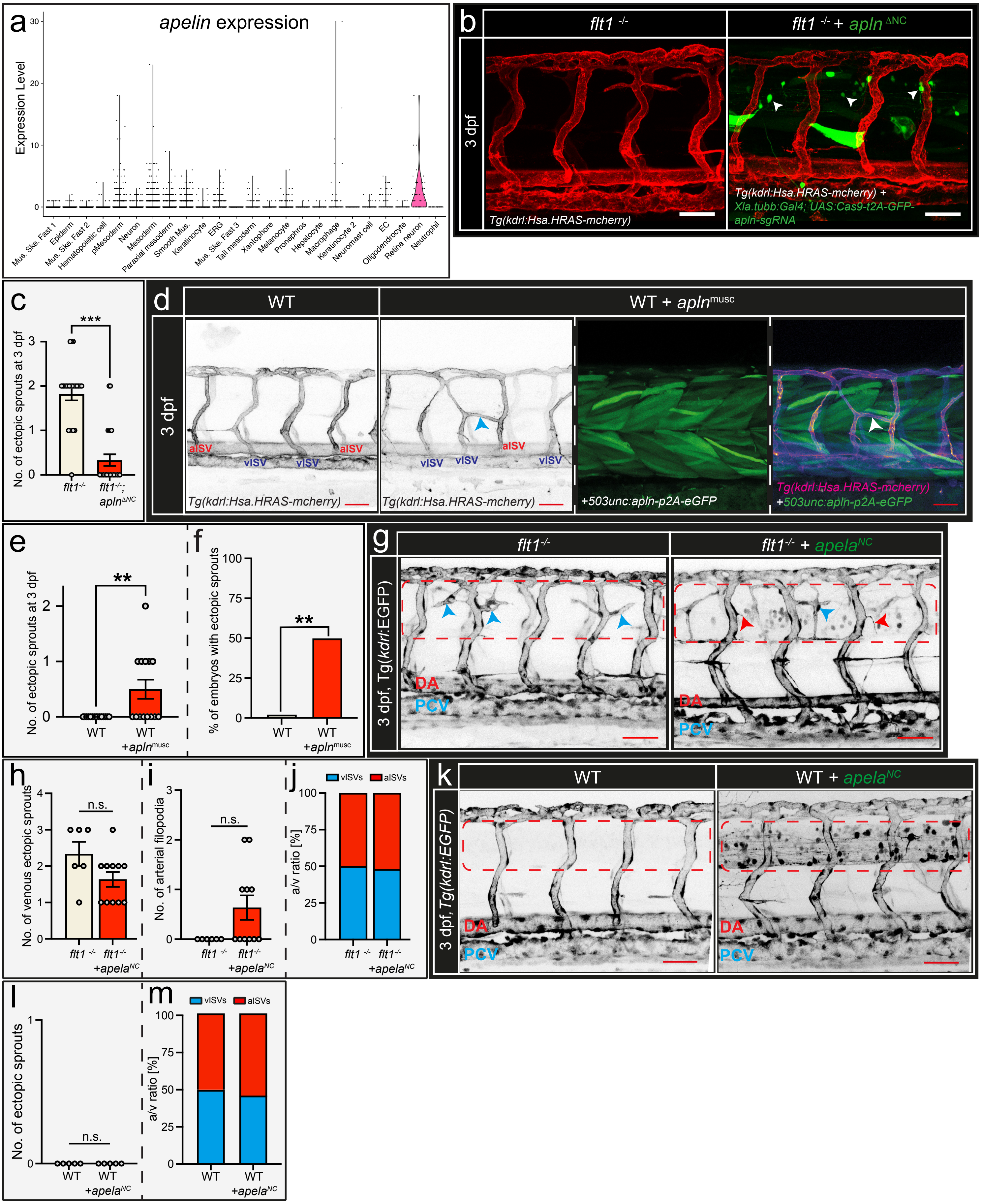
a
apelin expression
Expression Level
b
flt1 -/-
flt1 -/- + apln ΔNC
3 dpf
Tg(kdrl:Hsa.HRAS-mcherry)
Tg(kdrl:Hsa.HRAS-mcherry) +
Xla.tubb:Gal4; UAS:Cas9-t2A-GFP-
apln-sgRNA
c
No. of ectopic sprouts at 3 dpf
d
WT
WT + aplnmusc
aISV
vISV
Tg(kdrl:Hsa.HRAS-mcherry)
+503unc:apln-p2A-eGFP
e
No. of ectopic sprouts at 3 dpf
f
% of embryos with ectopic sprouts
g
flt1-/-
flt1-/- + apelaNC
3 dpf, Tg(kdrl:EGFP)
DA
PCV
h
No. of venous ectopic sprouts
n.s.
i
No. of arterial filopodia
j
vISVs
aISVs
a/v ratio [%]
k
WT
WT + apelaNC
l
No. of ectopic sprouts
m

Supplement Figure 10. Apelin/Aplnra signaling increases the Vegf/Kdrl signaling output

(**a**) Violin plot showing *apelin (apln)* expression for indicated cell types in zebrafish trunk.
(**b**) Confocal images of *Tg(kdrl:Hsa.HRAS-mCherry)* showing trunk vasculature in *flt1*$^{-/-}$ mutant (left panel), and *flt1*$^{-/-}$ mutant upon neural specific silencing of *apln* by co-expressing Cas9 and two apelin targeting *sgRNAs* under control of a neuronal specific promoter (*Xla.Tubb: Gal4; UAS: Cas9-apln-sgRNA* indicated as *apln*$^{\Delta NC}$) at 3 dpf (right panel, arrowheads, green cells express Cas9 and *sgRNAs*).
(**c**) Quantification of ectopic sprouts upon neuronal specific loss of apelin. Mean ± s.e.m., Two-sided Mann-Whitney U-test; *flt1*$^{-/-}$: n=23 embryos, *flt1*$^{-/-}$ + *apln*$^{\Delta NC}$: n=24 embryos.
(**d**) Confocal images of *Tg(kdrl:Hsa.HRAS-mCherry)* showing trunk vasculature in WT (left panel) and WT upon somite muscle specific overexpression of *apln* by injecting *503unc:apln-p2A-eGFP* expression construct, abbreviated *apln*^{musc} (right panels) at 3 dpf. Note: developing somites express *vegfaa*, and simultaneous co-expression of *apln* in somites affects patterning of the trunk vasculature.
(**e, f**) Quantification of ectopic branching (e) and phenotype frequency (f) for indicated scenarios. Mean ± s.e.m.; Two-sided Mann-Whitney U-test; WT: n=15 embryos and WT + *apln*^{musc}: n=14 embryos.
(**g**) Confocal images showing trunk vasculature in *flt1*$^{-/-}$ and *flt1*$^{-/-}$ mutant upon neuronal specific overexpression of *apela* by injection of a *Xla.tubb:EGFP-p2A-apela* construction (indicated as *apela*^{NC}) at 3 dpf. Red box indicates ROI; blue arrowheads indicate venous ectopic sprout and read arrowheads indicates arterial filopodia.
(**h, i, j**) Quantification of venous ectopic sprouts (h), arterial filopodia (i) and arterial to venous ISV ratio (j) for indicated scenario. Mean ± s.e.m. (h and i) or Mean (j); Two-sided Mann-Withney U test (h and i) or Fisher's test (j); *flt1*$^{-/-}$: n=6 embryos, *flt1*$^{-/-}$ + *apela*^{NC}: n=11embryos.
(**k**) Confocal images showing trunk vasculature in wildtype and wildtype upon neuronal specific overexpression of apela by injection of a *Xla.tubb:EGFP-p2A-apela* construction (indicated as apela^{NC}) at 3 dpf. Red box indicates ROI.
(l,m) Quantification of ectopic sprouts (l) and arterial to venous ISV ratio (m) for indicated scenario. Mean ± s.e.m. (l) or Mean (m); Two-sided Mann-Whitney U test (l) or Fisher's test (m); WT: n=5 embryos, WT + *apela*^{NC}: n=5 embryos.
Scale bar indicates 50 µm in b, d, g and k. NC, neuronal cell; EC, endothelial cell; SC, spinal cord; aISV, arterial intersegmental vessel; vISV, venous intersegmental vessel; musc, muscle; DA, dorsal aorta; PCV, posterior cardinal vein. *, $p<0.05$, **, $p<0.01$ and ***, $p<0.001$. Source data are provided as a Source Data file.

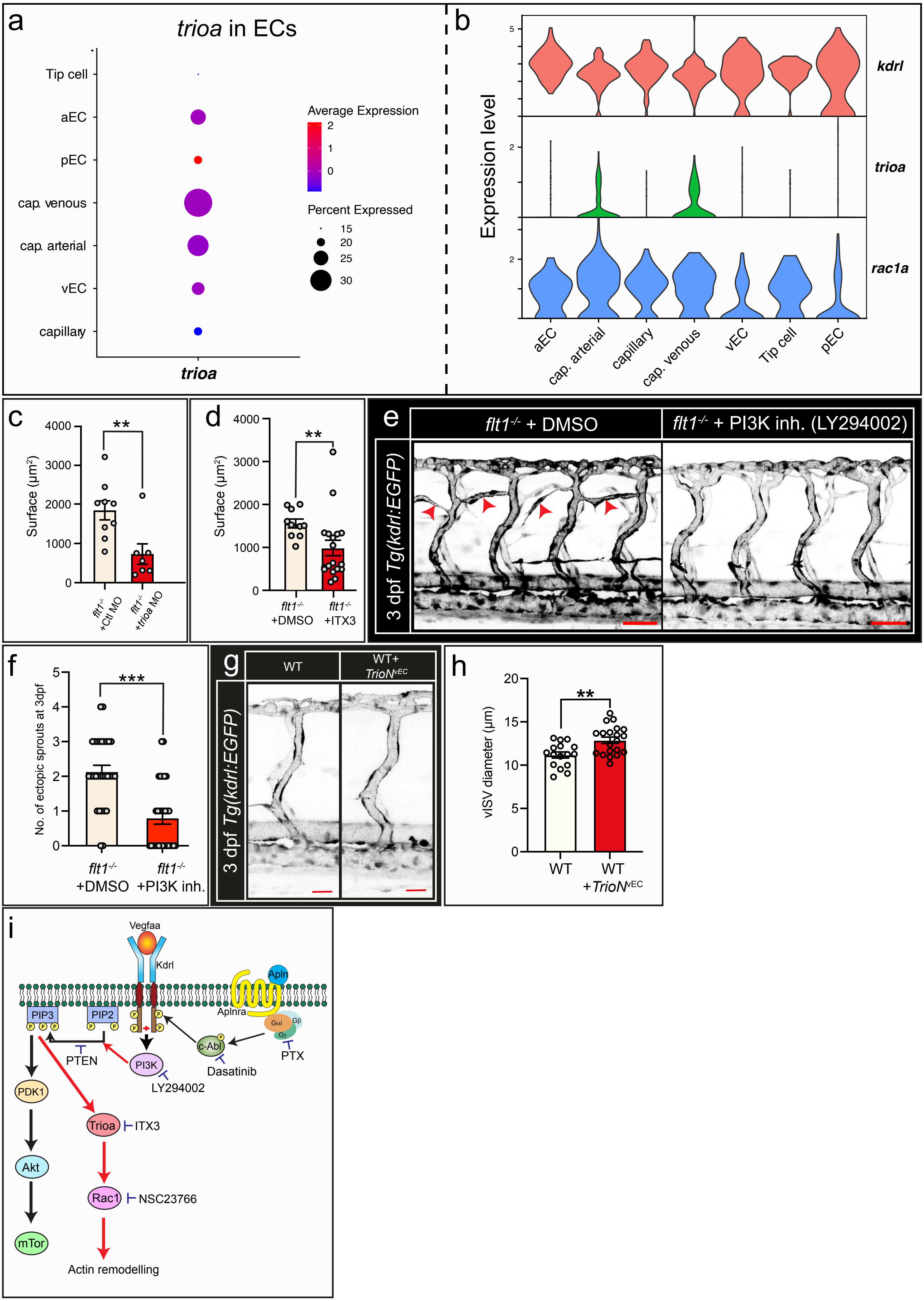
a
trioa in ECs
Tip cell
aEC
pEC
cap. venous
cap. arterial
vEC
capillary
trioa
Average Expression
Percent Expressed
b
Expression level
kdrl
trioa
rac1a
aEC
cap. arterial
capillary
cap. venous
vEC
Tip cell
pEC
c
Surface (µm²)
flt1-/- +Ctl MO
flt1-/- +trioa MO
d
flt1-/- +DMSO
flt1-/- +ITX3
e
flt1-/- + DMSO
flt1-/- + PI3K inh. (LY294002)
3 dpf Tg(kdrl:EGFP)
f
No. of ectopic sprouts at 3dpf
flt1-/- +DMSO
flt1-/- +PI3K inh.
g
WT
WT+ TrioNvEC
h
vISV diameter (µm)
WT +TrioNvEC
i
Vegfaa
Kdrl
Apln
Aplnra
PIP3
PIP2
PTEN
PI3K
LY294002
c-Abl
Dasatinib
PTX
PDK1
Trioa
ITX3
Akt
Rac1
NSC23766
mTor
Actin remodelling

Supplement Figure 11. Trio expression in endothelial cells

(**a**) Dot Plot showing *trioa* expression in endothelial cell subclusters.
(**b**) Violin Plot showing *kdrl*, *trioa* and *rac1a* expression in endothelial cell subclusters.
(**c**) Surface area of tertiary sprout network at the level of the spinal cord in *flt1*$^{-/-}$ and *flt1*$^{-/-}$ upon morpholino mediated knock-down of *trioa*. Mean ± s.e.m., Two-sided Mann-Whitney U test; Ctl MO: n=9 sprouts from 9 embryos, *trioa* MO: n=7 sprouts from 7 embryos.
(**d**) Surface area of tertiary sprout network at the level of the spinal cord in *flt1*$^{-/-}$ vehicle control and *flt1*$^{-/-}$ treated with Trio inhibitor ITX3. Mean ± s.e.m., Two-sided Mann-Whitney U test; DMSO, n=10 sprouts from 10 embryos, ITX: n=18 sprouts from 17 embryos.
(**e**) Confocal images of trunk vasculature in *flt1*$^{-/-}$ embryos treated with DMSO (left panel) or PI3K inhibitor (right panel) at 3 dpf. Red arrowheads indicate ectopic sprouts. Note: Inhibition of PI3K reduced tertiary sprout remodeling.
(**f**) Quantification based on images in (e). Note inhibition of PI3K reduced tertiary sprouting. Mean ± s.e.m.; Two-sided Mann-Whitney U test; n=30 embryos per conditions.
(**g**) Confocal images in *Tg(kdrl:EGFP)* showing vISV morphology in WT and WT injected with a *flt4:TrioN* expression construct to overexpress *Trio* in veins at 3 dpf. Note: *Trio* overexpression induces a vISV diameter increase but not ectopic sprouting.
(**h**) Quantification of vISV lumen diameter for indicated scenario. Mean ± s.e.m.; Two-sided Unpaired t-test; WT: n= 16 vISVs from 12 embryos, *flt4:TrioN*: n= 20 vISVs from 16 embryos.
(**i**) Schematic illustration of proposed coupling between Apelin-receptor-a and Kdrl *via* c-Abl, and activation of downstream pathways. The pathway interventions with pharmacological inhibitors (PTX, Dasatinib, LY294002, ITX3, NSC23766) are indicated.
Scale bar indicates 50 µm in e; 25 µm in g. aEC, arterial endothelial cell; vEC, venous endothelial cell; pEC, proliferating EC; cap, capillary; MO, morpholino; inh., inhibitor. *, p<0.05, **, p<0.01 and ***, p<0.001. Source data are provided as a Source Data file.

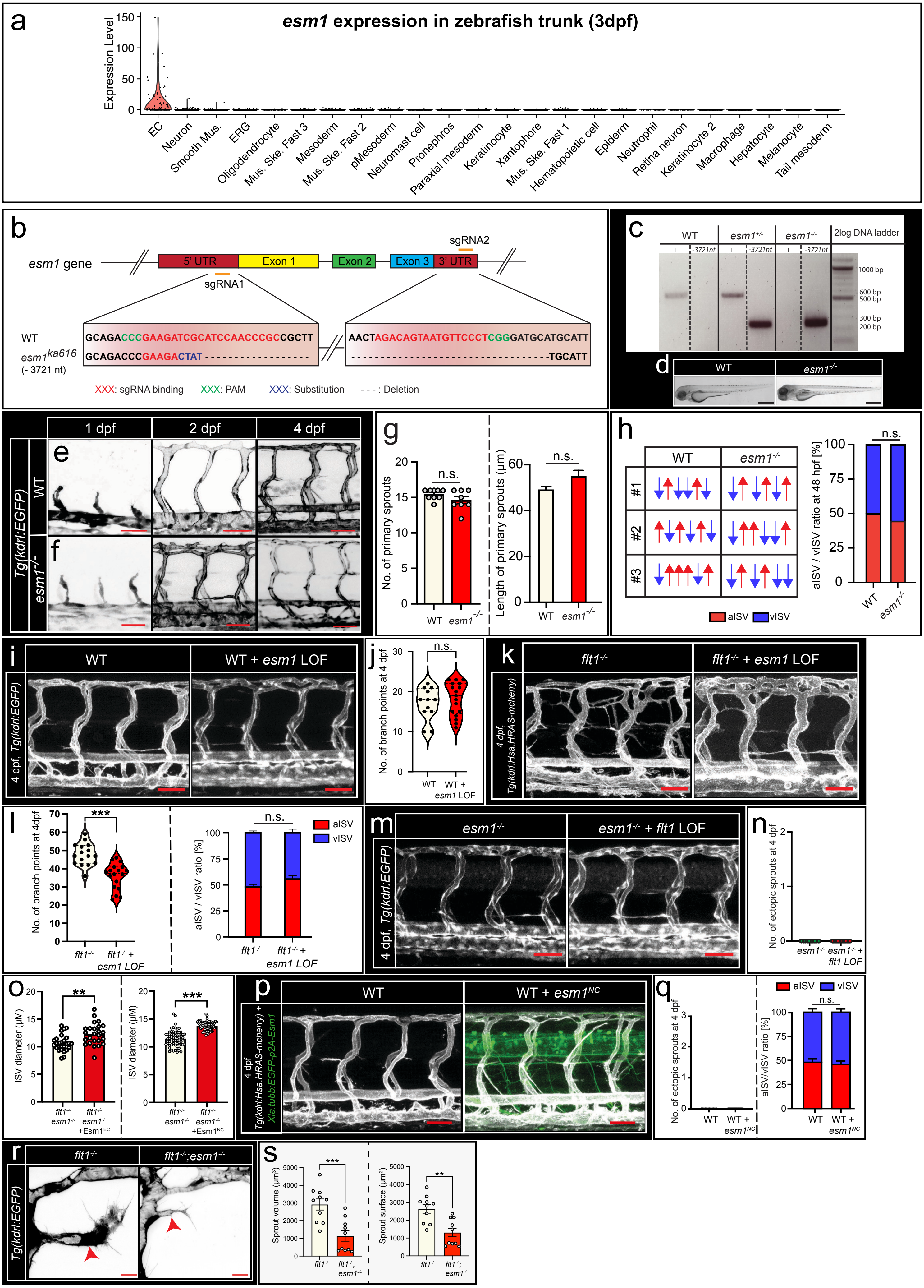
a
esm1 expression in zebrafish trunk (3dpf)
Expression Level
EC
Neuron
Smooth Mus.
ERG
Oligodendrocyte
Mus. Ske. Fast 3
Mesoderm
Mus. Ske. Fast 2
pMesoderm
Neuromast cell
Pronephros
Paraxial mesoderm
Keratinocyte
Xantophore
Mus. Ske. Fast 1
Hematopoietic cell
Epiderm
Neutrophil
Retina neuron
Keratinocyte 2
Macrophage
Hepatocyte
Melanocyte
Tail mesoderm
b
esm1 gene
5' UTR
Exon 1
Exon 2
Exon 3
3' UTR
sgRNA1
sgRNA2
WT
GCAGACCCGAAGATCGCATCCAACCCGCCGCTT
AACTAGACAGTAATGTTCCCTCGGGATGCATGCATT
esm1ka616
(- 3721 nt)
GCAGACCCGAAGACTAT
-TGCATT
XXX: sgRNA binding
XXX: PAM
XXX: Substitution
- - - : Deletion
c
WT
esm1+/-
esm1-/-
2log DNA ladder
-3721nt
1000 bp
600 bp
500 bp
300 bp
200 bp
d
WT
esm1-/-
1 dpf
2 dpf
4 dpf
Tg(kdrl:EGFP)
e
f
g
n.s.
No. of primary sprouts
Length of primary sprouts (µm)
WT
esm1-/-
h
#1
#2
#3
aISV
vISV
aISV / vISV ratio at 48 hpf [%]
i
WT
WT + esm1 LOF
4 dpf, Tg(kdrl:EGFP)
j
No. of branch points at 4 dpf
k
flt1-/-
flt1-/- + esm1 LOF
4 dpf, Tg(kdrl:Hsa.HRAS-mcherry)
l
***
No. of branch points at 4dpf
aISV / vISV ratio [%]
esm1 LOF
m
esm1-/-
esm1-/- + flt1 LOF
n
No. of ectopic sprouts at 4 dpf
flt1 LOF
o
**
ISV diameter (µM)
+Esm1EC
+Esm1NC
p
WT + esm1NC
4 dpf Tg(kdrl:Hsa.HRAS-mcherry) + Xla.tubb:EGFP-p2A-Esm1
q
aISV/vISV ratio [%]
r
flt1-/-;esm1-/-
s
Sprout volume (µm³)
Sprout surface (µm²)

Supplement Figure 12. Characterization of *esm1* mutant zebrafish.

(**a**) Violin plot showing *esm1* expression for indicated cell type in zebrafish trunk. Note: *esm1* is enriched in endothelial, neural and muscle cells.
(**b**) Exon-intron structure of the zebrafish *esm1* gene and binding sites for sgRNA1 in the 5' UTR region and sgRNA2 in the 3'UTR region. Cutting at both sites results in complete removal of the *esm1* open reading frame yielding $esm1^{ka616}$ mutant carrying a deletion of 3721 nucleotides.
(**c**) Amplicons of *esm1* heterozygous and homozygous mutant genotype compared to WT.
(**d**) *In vivo* light microscopy imaging of WT and $esm1^{-/-}$ mutant zebrafish embryo. $Esm1^{-/-}$ mutants showed no obvious abnormalities in morphogenesis.
(**e**) *In vivo* confocal images showing trunk vasculature in WT embryos at indicated time points.
(**f**) *In vivo* confocal images showing trunk vasculature in $esm1^{-/-}$ mutant embryos at indicated time points. $Esm1^{-/-}$ mutants look phenotypically normal and show no signs of aberrant vascular remodelling or ectopic sprouting.
(**g**) Quantification of primary sprouting events and sprout length in WT and $esm1^{-/-}$ mutant based on images in (e). Note: Loss of *esm1* had no measurable impact on the primary sprouting process. Mean ± s.e.m.; Two-sided Mann-Whitney U test; WT: n= 9 embryos, $esm1^{-/-}$: n= 8 embryos.
(**h**) Flow direction in intersegmental vessels and aISV/vISV ratio for indicated genotype and embryo (#1, #2, #3). Red arrow, arterial flow direction; blue arrow, venous flow direction. Percentage, Fisher's exact test; WT: n= 3 embryos, $esm1^{-/-}$: n= 3 embryos.
(**i**) Confocal images of trunk vasculature in WT injected with control (left panel), and *esm1* targeting morpholino (right panel) at 4 dpf.
(**j**) Quantification of vascular branching at ROI based on images in (**i**). Mean ± s.e.m., Two-sided Unpaired t-test, WT: n=12; *esm1* LOF: n=17.
(**k**) Confocal images of trunk vasculature in $flt1^{-/-}$ mutant injected with control (left panel), and *esm1* targeting morpholino (right panel) at 4 dpf.
(**l**) Quantification of vascular branching (left panel) and aISV to vISV ratio (right panel) based on images in (**k**). Mean ± s.e.m., Two-sided Unpaired t-test (left panel) and Fischer's test (right panel), $flt1^{-/-}$: n=16; $flt1^{-/-}$ + *esm1* LOF: n=18.
(**m**) Confocal images showing trunk vasculature in $esm1^{-/-}$ mutant injected with control (left panel) or *flt1* targeting morpholino (right panel) at 4 dpf.
(**n**) Quantification of ectopic sprouting based on images in (**m**). $esm1^{-/-}$: n=10 embryos, $esm1^{-/-}$ + *flt1 LOF*: n=14 embryos.
(**o**) Quantification of ISV diameter for indicated scenario. Note: Restoring vascular or neural *esm1* in $flt1^{-/-}$; $esm1^{-/-}$ double mutant induces a significant ISV diameter enlargement. Mean ± s.e.m., Two-sided Unpaired t-test with Welch's correction; left graph: n= 12-14 ISVs from 7 embryos per conditions, right graph: n= 65-67 ISVs from 18 and 20 embryos.
(**p**) Confocal images of *Tg(kdrl:Hsa.HRAS-mCherry)* showing trunk vasculature in WT (left panel) or WT injected with neuronal *esm1* gain of function construct ($esm1^{NC}$ right panel) at 4 dpf.
(**q**) Quantification of ectopic sprouting (left panel) and aISv/vISV ratio (right panel) based on images in (**p**). Left panel, scatter plot of individual values; Right panel: percentage, Mean ± s.e.m., Fischer's test. WT: n=12, WT+$esm1^{NC}$: n=18 embryos.
(**r**) Confocal images of *Tg(kdrl:EGFP)* reporter showing tertiary sprouts (red arrowhead) in $flt1^{-/-}$ (left) or $flt1^{-/-}$;$esm1^{-/-}$ double mutant (right) at 3 dpf. Note the reduced sprout size in $flt1^{-/-}$; $esm1^{-/-}$ mutants.
(**s**) Quantification of sprout volume (left) and surface (right) for the indicated scenarios. Mean ± s.e.m., Two-sided Unpaired t-test with Welch's correction (left) and Two-sided Mann Whitney (right). n= 10 embryos.
Scale bar indicates 500 µm in d; 50 µm in e, f, i, k, m, p; 10µm in r. EC, endothelial cell; Mus., Muscle; ERG, Ependymal radial glia; Ske., Skeletal; LOF, loss of function; NC, neuronal cell; GOF, gain of function. *, $p<0.05$, **, $p<0.01$ and ***, $p<0.001$. Source data are provided as a Source Data file.

Suppl. Fig. 13

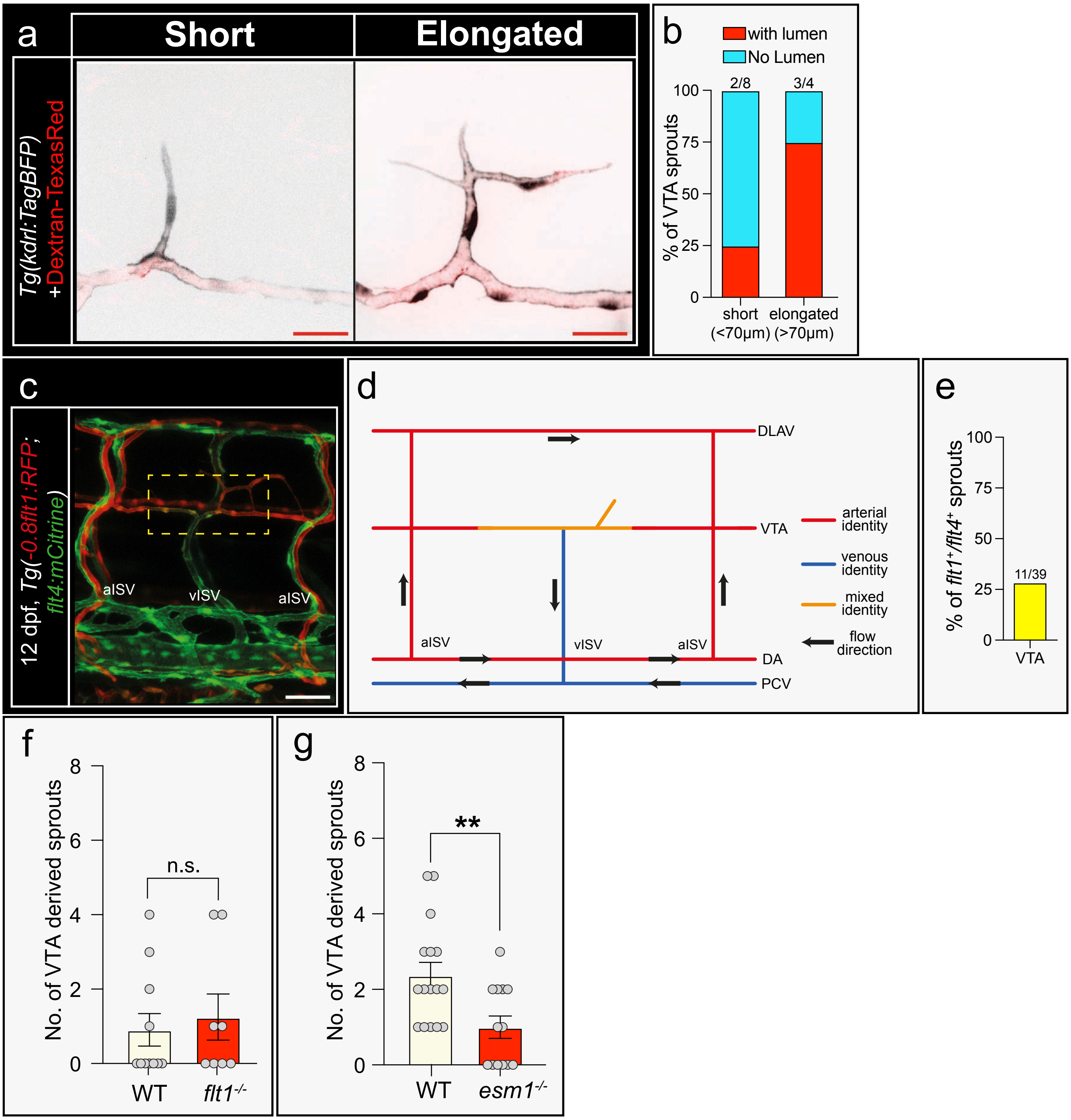
a
Short
Elongated
Tg(kdrl:TagBFP)
+Dextran-TexasRed
b
with lumen
No Lumen
2/8
3/4
% of VTA sprouts
100
75
50
25
0
short
(<70µm)
elongated
(>70µm)
c
12 dpf, Tg(-0.8flt1:RFP;
flt4:mCitrine)
aISV
vISV
aISV
d
DLAV
VTA
DA
PCV
aISV
vISV
aISV
arterial identity
venous identity
mixed identity
flow direction
e
% of flt1+/flt4+ sprouts
11/39
VTA
f
No. of VTA derived sprouts
n.s.
WT
flt1-/-
g
No. of VTA derived sprouts
**
WT
esm1-/-

Supplement Figure 13. Characterization of VTA-derived sprouts.

(**a**) Injection of TexasRed labelled dextran into *Tg(kdrl:TagBFP)* at 12 dpf to visualize blood plasma distribution and lumenization in VTA-derived sprouts. Short VTA sprouts (left) were predominantly not containing blood plasma indicating lack of lumenization; Elongated VTA sprouts (right) mostly contained blood plasma indicating of lumenization .
(**b**) Quantification of lumenization in VTA derived sprouts based on images in (a). n=12 VTA-derived sprouts.
(**c**) Confocal image of *Tg(-0.8flt1:RFP; flt4:mCitrine)* reporter showing the larvae trunk vasculature at 12 days. Note that in the VTA territory (boxed area), some segments expressed both the arterial marker *flt1* (red) and the venous marker *flt4* (green) suggesting mixed AV identity.
(**d**) Graphical illustration of the image in (c) showing the vessel's identities and the flow directions. The orange segment represents the portion of the VTA that expresses both arterial and venous markers suggestive of a population of endothelial cells with a mixed arterial-venous identity.
(**e**) Quantification of VTA-derived sprouts derived from *flt1/flt4* expressing VTA endothelium. Percentage, n=39 VTA-derived sprouts.
(**f**) Quantification of VTA-derived sprout number in WT siblings and *flt1*$^{-/-}$ mutants. Mean ± s.e.m., Two-sided Mann Whitney U test, WT: n=11; *flt1*$^{-/-}$: n=8.
(**g**) Quantification of VTA-derived sprout number in WT siblings and *esm1*$^{-/-}$ mutants. Mean ± s.e.m., Two-sided Mann Whitney U test, WT: n=16; *flt1*$^{-/-}$: n=13.
Scale bar indicates 20µm in a and 50µm in c. VTA, vertebral artery; aISV, arterial intersegmental vessel; vISV, venous intersegmental vessel. *, $p<0.05$, **, $p<0.01$ and ***, $p<0.001$. Source data are provided as a Source Data file.

Suppl. Fig. 14

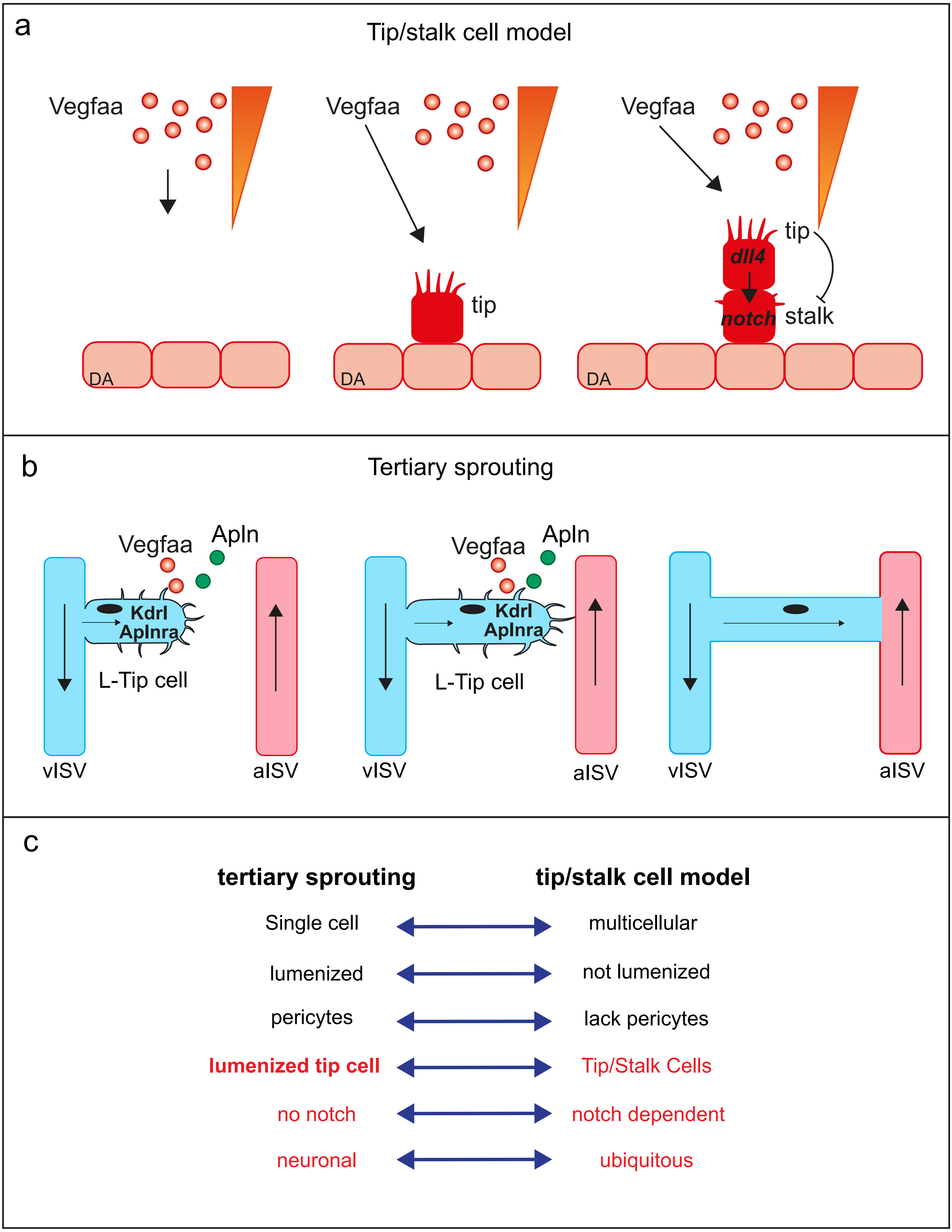
a
Tip/stalk cell model
Vegfaa
Vegfaa
Vegfaa
tip
tip
dll4
notch
stalk
DA
DA
DA
b
Tertiary sprouting
Vegfaa
Apln
Kdrl
Aplnra
L-Tip cell
vISV
aISV
Vegfaa
Apln
Kdrl
Aplnra
L-Tip cell
vISV
aISV
vISV
aISV
c
tertiary sprouting
tip/stalk cell model
Single cell
multicellular
lumenized
not lumenized
pericytes
lack pericytes
lumenized tip cell
Tip/Stalk Cells
no notch
notch dependent
neuronal
ubiquitous

Supplement Figure 14. Comparison of the Tip/stalk cell model with tertiary sprouting

(**a**) Schematic illustration of Vegfaa driven primary artery sprouting in the arterial domain - the reference sprouting model. In the arterial domain, Vegfaa induced sprout formation involves Dll4-Notch driven tip-stalk differentiation. Vegfaa-Kdrl signalling in the tip cell induces dll4 expression. Dll4 in the tip cell actives Notch receptors in the stalk cell. Activation of Notch results in translational activation of genes that reduce Kdrl responsiveness in stalk cells. This mechanism of lateral inhibition ensures that only one tip cell is formed. The tip cell is not lumenized, lumenization is a feature of the developing stalk cell.
(**b**) Schematic illustration of Vegfaa driven tertiary sprouting in the venous domain. Selection of the sprouting venous endothelial cell in the venous domain requires genetic interaction between Kdrl and Apelin-receptor-a signalling in venous endothelial cells. The ligands for these receptors, Vegfaa and Apelin respectively, are provided by neural cells located in the surrounding parenchyma. Tertiary sprouts consist of a single endothelial cell that enlarges, and forms a lumen – the Lumenized Tip-cell (L-Tip cell).
(**c**) Summary of the main differences between Vegf driven tertiary sprouting in the venous domain and Vegf driven sprouting in the arterial domain – the reference sprouting model.
DA, dorsal aorta; vISV, venous intersegmental vessel; aISV, arterial intersegmental vessel.

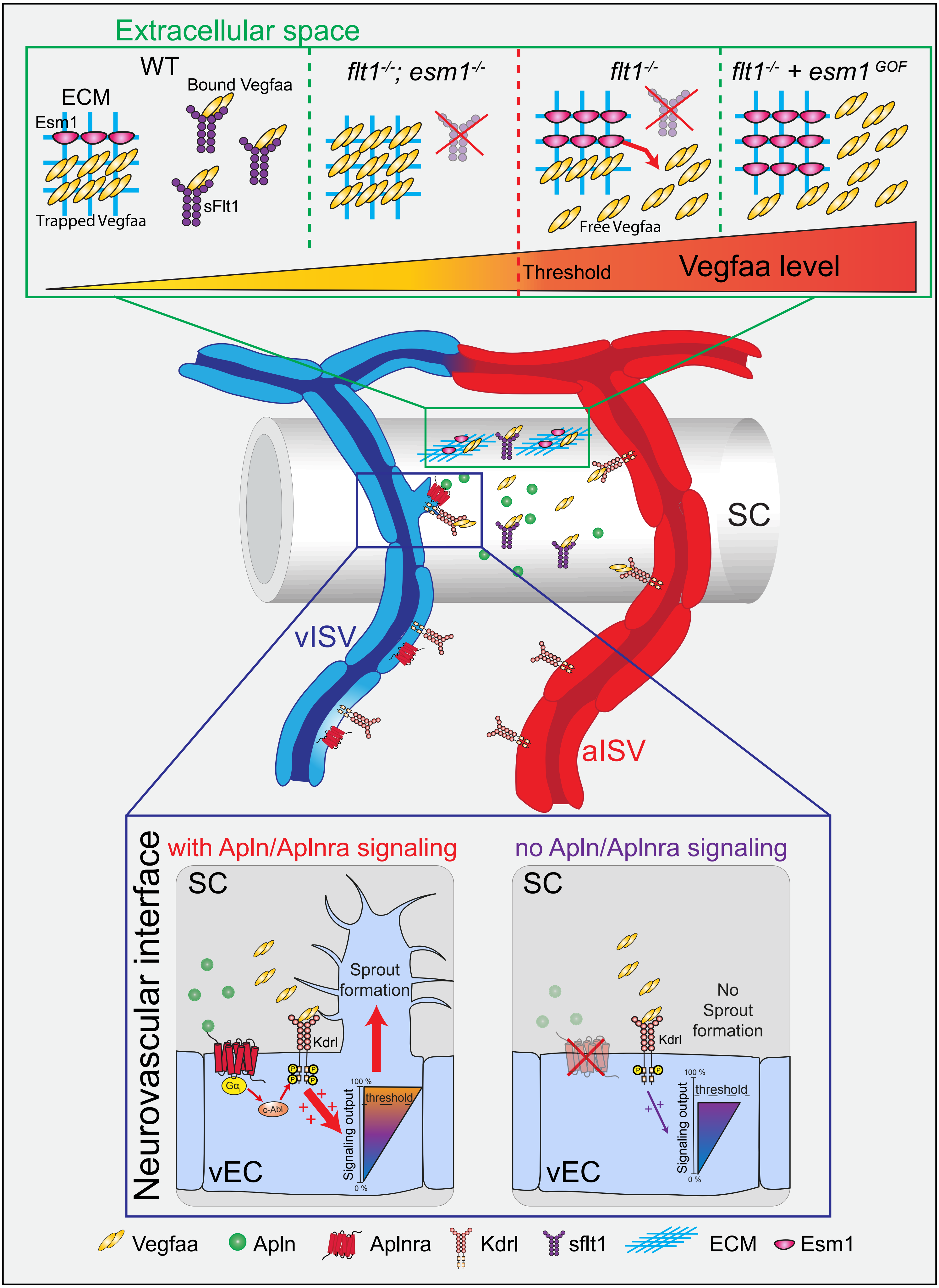
Extracellular space
WT
ECM
Esm1
Bound Vegfaa
sFlt1
Trapped Vegfaa
flt1-/-; esm1-/-
flt1-/-
Free Vegfaa
flt1-/- + esm1 GOF
Threshold
Vegfaa level
SC
vISV
aISV
Neurovascular interface
with Apln/Aplnra signaling
SC
Sprout formation
Kdrl
Gαi
c-Abl
100 %
threshold
Signaling output
0 %
vEC
no Apln/Aplnra signaling
SC
No Sprout formation
Kdrl
100 %
threshold
Signaling output
0 %
vEC
Vegfaa
Apln
Aplnra
Kdrl
sflt1
ECM
Esm1

Supplement Figure 15. Two-tiered regulation of tertiary sprouting.

Schematic illustration of Vegfa driven tertiary sprouting at the neuro-vascular interface in the trunk of the zebrafish embryo.

Top: regulation of Vegf at the level of the Parenchyma. In WT, Vegf is bound to either soluble Flt1 or the ECM, and the levels of free bio-available Vegf are low, and sprouting does not occur. In *flt1*$^{-/-}$ *;esm1*$^{-/-}$ double mutants, in the absence of *esm1* expression, Vegf initially trapped by soluble Flt1 now binds to the ECM. Vegf bio-availability levels remain low and consequently, sprouting is not induced. In *flt1*$^{-/-}$ mutants and in the presence of normal *esm1* expression levels, loss of soluble Flt1 (sFlt1) results in an increase of Vegf bio-availability and sprouting is induced. Esm1 gain of function results in displacement of Vegf from ECM binding moieties. Combining *esm1* gain of function with loss of sFlt1 (*flt1*$^{-/-}$ + *esm1*^{GOF}), results in high Vegf bio-availability and augments tertiary sprouting above the level observed in *flt1*$^{-/-}$ mutants with normal *esm1* expression levels.

Middle: interaction between parenchyma and the dorsal aspect of venous intersegmental vessels.

Bottom Left: cross-talk at the neuro-vascular interface in the presence of Apelin-receptor-a (Aplnra). Aplnra genetically interacts with Kdrl via the GαI domain and c-Abl kinase to enhance Kdrl signaling output above the threshold to induce tertiary sprouting. The ligands Apelin and Vegfa are provided by the neural cells of the surrounding parenchyma.

Bottom Right: in the absence of Aplnra, Kdrl signaling strength is not enhanced, stays below the threshold, and sprouting is not induced.

ECM, extracellular matrix; GOF, gain of function; SC, spinal cord; vISV, venous intersegmental vessel; aISV, arterial intersegmental vessel; vEC, venous endothelial cell.

Supplementary table 1: sgRNAs used to generate *esm1* mutant.

sgRNA name	Sequence (5'-3')
5' UTR sgRNA1	GAAGATCGCATCCAACCCGC
Exon 3 sgRNA2	AGACAGTAATAATGTTCCCT

Supplementary table 2: Primer for genotyping

Primer name	Sequence (5'-3')
Esm1_PCR1_fwd	CTGAGCCGCTTCATTCACTG
Esm1_PCR1_rev	TTATGCTTTAAATGTAGCACTCGAT
Esm1_PCR2_fwd	CTGAGCCGCTTCATTCACTG
Esm1_PCR2_rev	ATGGGATGCCATATCTGTGAACT
Aplnra_fwd	GCTGCCAGAGATTGGGATAA
Aplnra_rev	CGAGGTAGCGGTCAAAACTC
Aplnrb_fwd	CTGACCTGAGCACATGACAAA
Aplnrb_rev	CTGAGTGCTGGTCAGGGAGT
Flt1_fwd	ACACCTGAAGCATCTTACCTGTGA
Flt1_rev	CAGCTCAACACACACAGTATTGTTTTA

Supplementary table 3: Primer for p5E entry vector generation

Primer name	Sequence (5'-3')
mdk_flt4_fwd_SalI	CGCGTCGACAATTCCGCAGTCCGTCAATA
Mdk_flt4_rev_SmaI	CTACCCGGGCCTCCAGATCTCCAGTCCAG

Supplementary table 4: Primer for pME vector generation

Primer name	Sequence (5'-3')
Apln_fwd_AfeI	CGCAGCGCTATGAATGTGAAGATCTTGACG
Apln_rev_XbaI	CGATCTAGATGTCAAACTGACCTTGCT
Esm1 fwd	GCGCCCGGGCGTGTGTTTGCCATCCTG
Esm1 rev	ATTCTCGAGTCAGCGAGGGGTGAGG
Apela_SmaI_fwd	GAACCCGGGAGATTCTTCCACCCG
Apela_XhoI_rev	GGACTCGAGTCAAGGGAAAGGTACTC

Supplementary table 5: Primer for esm1 BAC recombineering

Primer name	Sequence (5'-3')
Esm1_HA1_control_fwd	CTGAGCCGCTTCATTCACTG
Esm1_HA1_control_rev	ATTTGTCCGGGCAATTCACG
Esm1_HA1_mCitrine_fwd	TCCTCCCCAACACTGCAGACCCGAAGATCGCATCCAACCCG CCGCTTGCAACCATGGTGAGCAAGGGCGAGGAG
Esm1_HA1_mCitrine_rev	GGACACGCTGAACTTGTGG
pTarBAC_HA1_iTol2_fwd	GCGTAAGCGGGGCACATTTCATTACCTCTTTCTCCGCACCCG ACATAGTCCCTGCTCGAGCCGGGCCCAAGTG
pTarBAC_HA1_iTol2_rev	GCGGGGCATGACTATTGGCGCGCCGGATCGATCCTTAATTA AGTCTACAATTATGATCCTCTAGATCAGATC
pTarBAC_HA1_Cntrl_fwd	CTGTCAAACATGAGAATTGGTC
pTarBAC_HA1_Cntrl_rev	GAGAGCCTTCAACCCAGTC

Supplementary table 6: Morpholinos

Target	Type	Sequence (5' -3')	amount [ng]	
apln	5' UTR	GATCTTCACATTCATTTCTGCTCTC	1	Scott et. al 2007
aplnra	5' UTR	TGTATTCCGACGTTGGCTCCATTTG	1	Scott et. al 2007
aplnrb	5' UTR	CAGAGAAGTTGTTTGTCATGTGCTC	1	Scott et. al 2007
cxcr4a	ATG	AGACGATGTGTTCGTAATAAGCCAT	1	Hollway et. al 2007
cxcl12a	ATG	ACTTTGAGTCCATGTTTGGCAGTG	1	Li et. al 2007
dll4	SB	TAGGGTTTAGTCTTACCTTGGTCAC	6	Geudens et. al 2010
flt1	ATG	ATATCGAACATTCTCTTGGTCTTGC	1	Krueger et. al 2011
trioa	5' UTR	AGCTCATGGCTGACGAAAAAACACA	0.7	Klems et. al 2020
control		CCTCTTACCTCAGTTACAATTTATA		GeneTools

Supplementary table 7: Inhibitors and chemicals

Name	Target	Final Concentration	Solvent	Manufacturer
AZ67	PFKB3	40 µM	DMSO	Sigma
CXCR4a Antagonist I	CXCR4a	25 µM	H_2O	Merck
Dasatinib	cAbl	4 µM	DMSO	Selleckchem
Olverembatinib	cAbl	10 uM	DMSO	Selleckchem
ITX3	Trio Gef1 domain	100 µM	DMSO	Sigma
NSC 23766	Rac1	100 µM	DMSO	Merck
LY294002	PI3K	5 µM	DMSO	Merck
DEXTRAN-FTIC		1 mg/ml	H_2O	Sigma
DMSO				Sigma

Supplementary table 8: qPCR primers

Target	Sequence (5' -3')
Trioa_fwd	ACATCGCCCTCATCATCAAACC
Trioa_rev	GCCGAGCATTGCCTGTAAAGTC
Apln_fwd	GCAGGGAAACGGAGGGAGG
Apln_rev	CTCTTTGCTATGCTCGGTGGAGG
Esm1_fwd	TTGTGACAGAGAAACCGGCG
Esm1_rev	AACCCACTTCATTACCTGCTTCA
Aplnra_fwd	GGACAAAACTCTGGGGGTGAA
Aplnra_rev	ACACTCGCATCCACTCATCG
Aplnrb_fwd	CCTCTTGCGCTATGGACTTC
Aplnrb_rev	GCCTGCAATCCAGTAGGTCT
b-actin_fwd	CGTGACATCAAGGAGAAGCT
b-actin_rev	TCGTGGATACCGCAAGATTC

References

1. Scott, I. C. *et al.* The G Protein-Coupled Receptor Agtrl1b Regulates Early Development of Myocardial Progenitors. *Dev Cell* **12**, 403–413 (2007).
2. Hollway, G. E. *et al.* Whole-Somite Rotation Generates Muscle Progenitor Cell Compartments in the Developing Zebrafish Embryo. *Dev Cell* **12**, 207–219 (2007).
3. Geudens, I. *et al.* Role of Delta-like-4/Notch in the Formation and Wiring of the Lymphatic Network in Zebrafish. *Arterioscler Thromb Vasc Biol* **30**, 1695–1702 (2010).
4. Krueger, J. *et al.* Flt1 acts as a negative regulator of tip cell formation and branching morphogenesis in the zebrafish embryo. *Development* **138**, 2111–2120 (2011).
5. Klems, A. *et al.* The GEF Trio controls endothelial cell size and arterial remodeling downstream of Vegf signaling in both zebrafish and cell models. *Nat Commun* **11**, (2020).
6. Li, Q. *et al.* Chemokine Signaling Guides Axons within the Retina in Zebrafish. *Journal of Neuroscience* **25**, 1711–1717 (2005).
