# Supplementary material for: Parenchymal cues define Vegfa-driven venous angiogenesis by activating a sprouting competent venous endothelial subtype
Source: Nat Commun. 2024 Apr 10;15:3118. doi: 10.1038/s41467-024-47434-x (PMC11006894; doi:10.1038/s41467-024-47434-x)
Supplement: Supplementary file 3 — Description of Additional Supplementary Files [file 41467_2024_47434_MOESM3_ESM.pdf]

Description of Additional Supplementary Files

File Name: Supplementary Movie 1
Description: Tertiary sprouts emanate from veins
Time-lapse imaging during tertiary sprout elongation and anastomosis formation in *Tg(-0.8flt1:RFP; flt4:mCitrine)*. Arteries are marked by *Tg(-0.8flt1:RFP) (red),* veins by *Tg(flt4:mCitrine) (green)*. Time-lapse from 80 hpf to 99.5 hpf at 2 frames per hour. Scale bar represents 20µm.

File Name: Supplementary Movie 2
Description: Ventral-dorsal migration of EC in venous ISVs of WT
Time-lapse imaging of *Tg(kdrl:Hsa.HRAS-mCherry; fli1:nEGFP)* reporter showing venous endothelial cell movements in WT between 55 hpf and 73 hpf. 3 frames per hour. Arrowheads indicate individual vEC nuclei. Scale bar represents 20µm.

File Name: Supplementary Movie 3
Description: Ventral-dorsal migration of EC in venous ISVs of *flt1*$^{-/-}$
Time-lapse imaging of *Tg(kdrl:Hsa.HRAS-mCherry; fli1:nEGFP)* reporter showing venous endothelial cell movements in *flt1*$^{-/-}$ between 55 hpf and 73 hpf. 3 frames per hour. Arrowheads indicate individual vEC nuclei. Scale bar represents 20µm.

File Name: Supplementary Movie 4
Description: Ventral-dorsal migration of venous and arterial EC in vISVs of *flt1*$^{-/-}$
Time-lapse imaging of *Tg(-0.8flt1:RFP; flt4:mCitrine)* during venous remodelling process in *flt1*$^{-/-}$ mutant between 49 hpf and 66 hpf. 2 frames per hour. Venous endothelial cells (green) migrate from the PCV into the venous ISV and displace the arterial endothelial cells (red). Scale bar represents 20µm.

File Name: Supplementary Movie 5
Description: Ventral-dorsal migration of venous and arterial EC in vISVs of *flt1*$^{-/-}$ injected with *aplnra* morpholino.
Time-lapse imaging of *Tg(-0.8flt1:RFP; flt4:mCitrine)* during venous remodelling process in *flt1*$^{-/-}$ with *aplnra* loss of function between 49 hpf and 66 hpf. 2 frames per hour. Venous endothelial cells (green) have reduced migration and are not displacing the arterial endothelial cells (red). Scale bar represents 20µm.
